# Water in Solvate Ionic Liquids: Preserving Lithium Coordination While Enhancing Ionic Conductivity

**DOI:** 10.1002/cphc.70353

**Published:** 2026-04-17

**Authors:** Jule Kristin Philipp, Dietmar Paschek, Lennart Kruse, Annette‐Enrica Surkus, Bennet Austrup, Monika Schönhoff, Ralf Ludwig

**Affiliations:** ^1^ Institut für Chemie, Physikalische und Theoretische Chemie Universität Rostock Rostock Germany; ^2^ Leibniz‐Institut für Katalyse (LIKAT) an der Universtät Rostock Rostock Germany; ^3^ Institute of Physical Chemistry University of Münster Münster Germany; ^4^ Department LL&M Universität Rostock Rostock Germany

**Keywords:** cluster formation, molecular dynamics simulations, solvate ionic liquid, structure, water‐in‐salt electrolyte

## Abstract

This work investigates how the concepts of solvate ionic liquids (SILs) and water‐in‐salt (WIS) electrolytes can be combined to create hybrid electrolyte systems. We examine the neat SIL [Li(G3)][NTf_2_], composed of a solvate cation and an anion, as well as its water‐modified analog containing an equimolar amount of added water, using both molecular dynamics simulations and experiments. Introducing water markedly reduces the high viscosity of the neat SIL while substantially enhancing ionic conductivity. Structurally, each cationic complex incorporates on average a single water molecule, resulting in highly dispersed water and the absence of extended water networks. We refer to such systems as *water‐in‐solvate‐ionic‐liquid* (WISIL) electrolytes. Owing to the strongly coordination‐dominated lithium environment, the WISIL retains a wide electrochemical stability window, decreasing only slightly from over 5 V in the neat SIL to 4.9 V at ambient conditions.

## Graphical abstract

By adding a controlled amount of water to a solvate ionic liquid, this concept can be bridged with water‐in‐salt electrolytes. Water is inserted into the characteristic cationic complex, leading to water‐in‐solvate‐ionic‐liquid electrolytes.

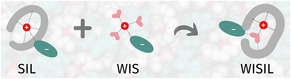

## 1 Introduction

Solvate ionic liquids (SILs) represent a distinct class of liquid electrolytes composed of a salt and a molecular solvent capable of forming a stable chelate complex depending on the mixing ratio [1, 2, 3, 4]. This unique coordination gives rise to physicochemical properties comparable to those of conventional ionic liquids (ILs) [5, 6, 7]. In addition, SILs exhibit remarkably high thermal stability, further broadening their applicability in electrochemical systems [4]. By tailoring the composition of a SIL, these properties can be finely tuned, making them promising candidates for safer and more environmentally friendly battery electrolytes [6, 7, 8, 9].

A well‐studied class of SILs is equimolar mixtures of lithium bis(trifluoromethanesulfonyl)imide ([Li][NTf_2_]) with glyme solvents (H–(CH_2_–O–CH_2_)*_n_*–H) such as triglyme (G3, *n* = 4) [6]. Their distinct coordination chemistry leads to well‐defined complex cations, e.g., [Li(G3)]^+^, which exhibit high thermal and electrochemical stability. Nevertheless, the high viscosities and correspondingly low ionic conductivities of these systems limit their practical applicability [4, 5, 6].

Previous studies have shown that dilution with molecular solvents can substantially enhance the ionic conductivity of SILs by lowering viscosity [6, 10, 11, 12]. However, the nature of the additive plays a crucial role. Nonpolar solvents primarily act as viscosity modifiers without significantly destabilizing the cation–glyme complexes. In contrast to this, Ueno et al. demonstrated that highly polar solvents, such as water, can interfere with the [Li]^+^ solvation environment by competing with glyme ligands for coordination [10, 12, 13].

Despite these challenges, aqueous electrolytes remain attractive due to their inherent safety and sustainability. However, their narrow electrochemical stability window (ESW) limits their applicability in systems operating at the electrochemical potentials of conventional Li‐ion battery electrodes [10, 14]. To overcome these limitations, a new class of electrolytes, water‐in‐salt (WIS) systems, has recently emerged. In these highly concentrated aqueous electrolytes, where the salt content exceeds that of water in both mass and volume, lithium cations remain closely associated with their counterions rather than being fully hydrated. Such water‐rich yet ion‐dense systems exhibit remarkably broad ESWs of up to ≈ 3V at room temperature, compared to 1.23 V for conventional aqueous electrolytes, due to the formation of stable interphases that suppress water reduction [15, 16, 17, 18, 19, 20].

In this work, we aim to combine the advantages of SIL and WIS electrolytes by introducing an equimolar amount of water into the SIL [Li(G3)][NTf_2_]. Specifically, we address three key questions: (1) How does the addition of water influence the transport and electrochemical properties of the SIL? (2) How does water affect the local solvation structure of lithium cations? and (3) What specific role does water play at such low concentrations in determining the overall physicochemical behavior of the electrolyte?

## 2 Computational and Experimental Methods

To address these questions, we investigated the neat SIL ([Li][NTf_2_]:G3:H_2_O = 1:1:0) and its water‐modified analog containing an equimolar amount of water ([Li][NTf_2_]:G3:H_2_O = 1:1:1) using both experiments and molecular dynamics (MD) simulations at 303 and 323 K. Two MD setups were employed: initial simulations in a cubic box, followed by multimicrosecond orthorhombic simulations using the OrthoBoXY approach by Busch and Paschek to determine system‐size independent self‐diffusion coefficients and viscosities [21, 22]. The simulations were complemented by experimental measurements of transport properties, including self‐diffusion coefficients from pulsed‐field gradient NMR [23] and electrochemical behavior from cyclic voltammetry (CV). Detailed descriptions of all experimental procedures and simulation protocols are provided in the Supporting Information (SI).

Experimental densities are well reproduced by the MD simulations for both compositions and temperatures (see SI), providing confidence in the employed molecular model and the microscopic interpretation developed in this study.

## 3 Results and Discussion

**FIGURE 1:**
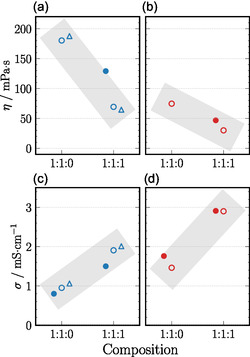
Shear viscosity η (a,b) and ionic conductivity σ (c,d) obtained from experiment (open circles) as well as MD simulations (filled circles) at 303 K (blue) and 323 K (red). Mixture compositions 1:1:0 and 1:1:1 correspond to [Li][NTf_2_]:G3:H_2_O. Experimental reference data at 303 K (open triangles) are taken from refs. [10, 24]. Gray bars serve as visual guides to highlight trends; individual data points are horizontally offset for clarity.

Figure 1 summarizes the experimental and simulated shear viscosities and ionic conductivities of both systems at the two investigated temperatures. The addition of water has a pronounced effect on the shear viscosity. The neat SIL exhibits a high viscosity of 180 mPa s at 303 K, which decreases by more than half upon heating to 323 K. Introducing water substantially lowers the viscosity to 69 mPa s at 303 K and to 30 mPa s at 323 K. Viscosities obtained from MD simulations are of the same order of magnitude as the experimental values. However, the high viscosities necessitate long simulation trajectories to achieve acceptable statistical accuracy. Although multimicrosecond simulations still lead to noticeable deviations from experiment, the simulations reproduce the experimental trends well.

The ionic conductivity follows the opposite trend. The neat SIL shows a low conductivity of 0.95 mS cm^−1^ at 303 K, nearly an order of magnitude smaller than that of conventional electrolytes [25]. The water‐modified system, however, exhibits an approximately twofold increase in conductivity. At 323 K, the conductivity rises from 1.46 to 2.90 mS cm^−1^, in near‐quantitative agreement with MD simulations. At 303 K, simulations slightly underestimate the conductivity, likely due to the limited ion mobility and associated statistical uncertainty in this highly viscous regime.

Overall, the experimental and simulated results are in very good agreement across the measured properties. This consistency demonstrates that the molecular models accurately capture the macroscopic behavior of these complex electrolytes and supports the reliability of the simulated structural motifs in representing the real liquid systems.

**FIGURE 2:**
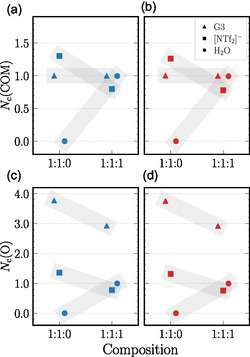
Coordination numbers of [Li]^+^ with G3 (triangles), [NTf_2_]^−^ (squares), and H_2_O (circles) from MD simulations at 303 K (blue) and 323 K (red). $N_{c}$(COM) (a,b) denotes the average number of molecules coordinated to [Li]^+^ based on center‐of‐mass (COM) distances, while oxygen coordination numbers (c,d) represent atomic‐level contributions. Mixture compositions 1:1:0 and 1:1:1 correspond to [Li][NTf_2_]:G3:H_2_O. Gray bars serve as visual guides to highlight trends; individual data points are horizontally offset for clarity.

**FIGURE 3:**
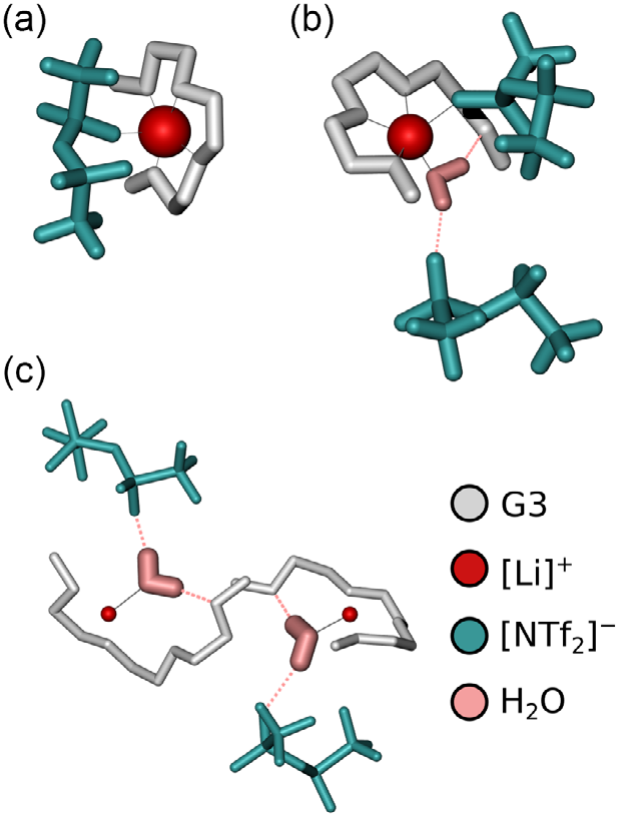
Typical lithium environment for the mixtures with the [Li][NTf_2_]:G3:H_2_O composition of (a) 1:1:0 (neat SIL) and (b) 1:1:1, while (c) highlights the spatial separation of water molecules due to the incorporation in complex cations in the water‐modified SIL. All snapshots are taken from the MD simulations at 303 K. [NTf_2_]^−^ anions, [Li]^+^ cations, G3 molecules, and H_2_O molecules are depicted in cyan, red, gray, and pink, respectively. Coordinative bonds are illustrated by black, solid lines, while HBs are shown as pink, dashed lines.

Figure 2 provides insights into the local coordination environment of the lithium cations at both investigated temperatures. Overall, both systems exhibit similar lithium coordination numbers within the studied temperature range, as derived from the data in Table S5. As shown in a previous study, the characteristic coordination motif of the neat SIL remains largely unchanged up to 483 K [4]. In the binary, water‐free system, each lithium cation is on average coordinated by a single triglyme molecule, whose four oxygen atoms form a stable chelate complex. The coordination sphere is further complemented by anions, with one or two [NTf_2_]^−^ anions contributing one oxygen atom each. A representative snapshot of the most abundant structural motif in the neat SIL is shown in Figure 3a.

Upon addition of an equimolar amount of water, the coordination environment of lithium undergoes subtle but distinct changes. Most lithium cations remain associated with a single triglyme molecule; however, in the majority of cases, only three of its oxygen atoms now directly participate in coordination. The fourth site—most likely one of the two terminal oxygen atoms of the G3 molecule (see Table S9 and Figure S6)—is replaced by a water molecule. This water molecule enters the first coordination shell and simultaneously forms hydrogen bonds (HBs) with both the displaced triglyme oxygen and a nearby anion (Figure 3b). Furthermore, the average number of anions in the first solvation shell decreases to below one, with some anions partially replaced by water. As a result, the overall lithium–oxygen coordination number decreases from 5.1 to 4.7. Additional details on the distribution of structural motifs surrounding [Li]^+^ are provided in the SI.

**FIGURE 4:**
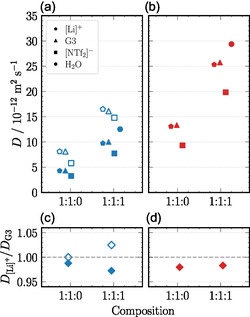
Self‐diffusion coefficients of [Li]^+^ (pentagons), G3 (triangles), [NTf_2_]^−^ (squares), and H_2_O (circles) from MD simulations (filled symbols) at (a) 303 K (blue) and (b) 323 K (red). At 303 K, experimentally obtained values are given as open symbols. (c) and (d) Ratio of self‐diffusion coefficients of [Li]^+^ and G3 obtained from MD simulations (closed symbols) and experiment (open symbols) at 303 K (blue diamonds) and 323 K (red diamonds), respectively. Mixture ratios of 1:1:0 and 1:1:1 correspond to [Li][NTf_2_]:G3:H_2_O, respectively. Individual data points are horizontally offset for clarity.

The stability of such complex cations can be assessed by comparing the self‐diffusion coefficients of the lithium cation and the coordinating glyme. The ratio $D_{{\left[\mathrm{Li}\right]}^{+}}/D_{G3}$ reflects whether lithium and G3 diffuse together, with values near unity indicating stable cationic complexes [5, 12]. As shown in Figure 4c,d, this ratio remains close to unity for both the neat SIL and the water‐modified SIL at 303 and 323 K in experiment and simulation. The underlying self‐diffusion coefficients are demonstrated in Figure 4a for 303 K and in Figure 4b for 323 K. Although experimentally measured self‐diffusion coefficients are generally enhanced by a factor of ≈2 compared to MD simulation results, they increase with temperature and are consistently higher in the water‐containing system, reflecting the reduced viscosity. Yet, the relative mobilities of [Li]^+^ and G3 remain similar. This finding demonstrates that adding an equimolar amount of water does not significantly disrupt the integrity of the complex cations. While the chosen classical force fields reproduce the trends well, we note that polarizable models are increasingly available and offer promising improvements in accuracy, particularly for ILs [26], and might therefore result in better quantitative agreement of simulation and experiment.

The incorporation of water into the lithium coordination sphere results in the effective spatial separation and dispersion of individual water molecules throughout the system. This behavior is shown in Figure 3c, where neighboring complex cations each contain a single, internally coordinated water molecule that is sterically hindered from forming water–water HBs. Consequently, only about 1.8 % of the water molecules engage in such interactions at both 303 and 323 K (see SI for more details on computation).

Because the characteristic structural motif of the 1:1:1 system combines features of both disrupted water networks typical of WIS electrolytes and the stable chelate complexes characteristic of SILs, we introduce the term *water‐in‐solvate‐ionic‐liquid* (WISIL) electrolyte for this water‐modified SIL.

At both temperatures, more than 99% of all triglyme and water molecules participate in lithium coordination. This coordination‐dominated environment is advantageous in SILs, as it minimizes the amount of free solvent and thereby reduces solvent degradation at the electrodes [7]. The absence of bulk‐like water clusters and the extremely low fraction of uncoordinated solvent molecules suggest an ESW wider than that of conventional aqueous electrolytes and comparable to WIS systems.

**FIGURE 5:**
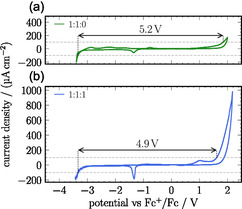
CV curves of (a) the neat SIL (green, measured at 303 K) as well as (b) with an equimolar amount of water added (blue, measured at 296 K). The respective ESWs are denoted above each CV curve. Gray‐dashed lines mark the limits for determining the respective ESWs at −0.1 and 0.1 mA cm^−2^. Mixture ratios of 1:1:0 and 1:1:1 correspond to [Li][NTf_2_]:G3:H_2_O, respectively.

CV measurements (see Figure 5) confirm this. The broad ESW, determined at the current density limits of ±0.1 mA cm^−2^, of the neat SIL of 5.2 V is only slightly reduced to 4.9 V in the WISIL. The 1:1:1 WISIL, however, shows a minor anodic peak at 1.03–1.07 V versus Fc^+^/Fc attributed to the oxidation of trace amounts of free water at the anode. In addition, a distinct cathodic peak at −1.33 V versus Fc^+^/Fc emerges. Following Suo et al. and in analogy to classical WIS electrolytes, this cathodic feature is likely indicative of the formation of a solid electrolyte interphase (SEI) [15, 17]. In WIS electrolytes, cathodic reduction of residual surface water molecules generates a locally alkaline environment near the electrode, which promotes anion reduction at potentials close to the hydrogen evolution reaction. The resulting passivating SEI significantly extends the anodic limit of the ESW compared with conventional aqueous electrolytes. A very small cathodic feature at the same potential can also be discerned in the neat SIL, which may also originate from SEI formation. However, further surface analysis is required to confirm the composition of the passivating SEI.

## 4 Conclusions

To conclude, experimental data and MD simulations consistently show that adding an equimolar amount of water to the neat SIL [Li(G3)][NTf_2_] improves the transport properties of the modified SIL by reducing viscosity and thereby doubling ionic conductivity. Structurally, lithium remains coordinated by one G3 molecule, with a single water molecule substituting one donor site and forming HBs to the G3 ligand and nearby anions. Despite this modification, the self‐diffusion coefficient ratio of [Li]^+^ and G3 remains close to unity, confirming the persistence of long‐lived complex cations. The dispersed, nonassociating water molecules enhance transport without disrupting the SIL structure, acting as a molecular lubricant, while maintaining a broad ESW, probably due to the formation of a passivating SEI. Previous studies have shown, however, that the beneficial effects are lost when larger amounts of water are introduced, leading to the breakdown of the complex structure [10]. These insights highlight a narrow but valuable compositional window for optimizing SIL‐based electrolytes and motivate further studies toward fine‐tuning solvation structure and ion mobility for next‐generation electrolytes.

## Supporting Information

Additional supporting information can be found online in the Supporting Information section.

## Author Contributions

**Jule Kristin Philipp**: conceptualization, methodology, data curation, investigation, validation, formal analysis, visualization, writing – original draft, writing – review & editing, software. **Dietmar Paschek**: conceptualization, methodology, data curation, investigation, project administration, writing – review & editing, software, writing – original draft. **Lennart Kruse**: investigation, writing – review & editing, data curation, writing – original draft. **Annette‐Enrica Surkus**: investigation, writing – review & editing, writing – original draft. **Bennet Austrup**: investigation, writing – original draft. **Monika Schönhoff**: writing – review & editing, supervision, validation, resources. **Ralf Ludwig**: supervision, funding acquisition, writing – original draft, writing – review & editing, conceptualization, project administration, methodology, resources.

## Funding

This work was supported by the Deutsche Forschungsgemeinschaft (LU‐506/17‐1 (project no. 470038970) and LU‐506/18‐1 (project no. 517661181)).

## Conflicts of Interest

The authors declare no conflicts of interest.

## Supporting information

Supplementary Material

## Data Availability

The code of GROMACS is freely available. Input parameter and topology files for the MD simulations can be downloaded from GitHub via https://github.com/Paschek‐Lab/WISIL‐G3/.
