# Supplementary material for: Water in Solvate Ionic Liquids: Preserving Lithium Coordination While Enhancing Ionic Conductivity
Source: Chemphyschem. 2026 Apr 17;27(8):e70353. doi: 10.1002/cphc.70353 (PMC13090136; doi:10.1002/cphc.70353)
Supplement: Supplementary file 1 — Supplementary Material [file CPHC-27-e70353-s001.pdf]

Research Article

Water in Solvate Ionic Liquids: Preserving Lithium Coordination While Enhancing Ionic Conductivity

Submission ID d0e3e002-1610-4cf5-bd9b-e38f736f66c5

Submission Version Revision 1

PDF Generation 20 Feb 2026 11:12:54 EST by Atypon ReX

Authors

Ms. Jule Kristin Philipp

ORCiD
https://orcid.org/0000-0002-9253-3239

CRediT
Conceptualization, Methodology, Data curation, Investigation, Validation, Formal analysis, Visualization, Writing - original draft, Writing - review & editing, Software

Affiliations
- Institut für Chemie, Physikalische und Theoretische Chemie, Universität Rostock, Albert-Einstein-Straße 27, D-18059 Rostock, Germany

Dr. Dietmar Paschek

ORCiD
https://orcid.org/0000-0002-0342-324X

CRediT
Conceptualization, Methodology, Data curation, Investigation, Project administration, Writing - review & editing, Software, Writing - original draft

Affiliations
- Institut für Chemie, Physikalische und Theoretische Chemie, Universität Rostock, Albert-Einstein-Straße 27, D-18059 Rostock, Germany

Mr. Lennart Kruse

ORCiD
https://orcid.org/0000-0002-8631-1840

CRediT
Investigation, Writing - review & editing, Data curation, Writing - original draft

Affiliations
- Institut für Chemie, Physikalische und Theoretische Chemie, Universität Rostock, Albert-Einstein-Straße 27, D-18059 Rostock, Germany

Mrs. Annette-Enrica Surkus

ORCiD
https://orcid.org/0000-0003-1161-9848

CRediT
Investigation, Writing - review & editing, Writing - original draft

Affiliations

- Leibniz-Institut für Katalyse (LIKAT) an der Universtät Rostock, Albert-Einstein-Straße 29a, D-18059 Rostock, Germany

Mr. Bennet Austrup

ORCiD
https://orcid.org/0009-0001-6396-4953

CRediT
Investigation, Writing - original draft

Affiliations

- Institute of Physical Chemistry, University of Münster, Corrensstr. 28/30, D-48149 Münster, Germany

Prof. Monika Schönhoff

ORCiD
https://orcid.org/0000-0002-5299-783X

CRediT
Writing - review & editing, Supervision, Validation, Resources

Affiliations

- Institute of Physical Chemistry, University of Münster, Corrensstr. 28/30, D-48149 Münster, Germany

Prof. R. Ludwig
Corresponding Author
Submitting Author

ORCiD
https://orcid.org/0000-0002-8549-071X

CRediT
Supervision, Funding acquisition, Writing - original draft, Writing - review & editing, Conceptualization, Project administration, Methodology, Resources

Affiliations

- Institut für Chemie, Physikalische und Theoretische Chemie, Universität Rostock, Albert-Einstein-Straße 27, D-18059 Rostock, Germany
- Leibniz-Institut für Katalyse (LIKAT) an der Universtät Rostock, Albert-Einstein-Straße 29a, D-18059 Rostock, Germany
- Department LL&M, Universität Rostock, Albert-Einstein-Straße 25, D-18059 Rostock, Germany

Files for peer review

All files submitted by the author for peer review are listed below. Files that could not be converted to PDF are indicated; reviewers are able to access them online.

Name	Type of File	Size	Page
20260220_reviewer_response.pdf	Author Response	106.8 KB	Page 4
20260220_paper_revision.pdf	Main Document - LaTeX PDF	1.8 MB	Page 9
20260220_SI_revised.pdf	Supporting Information for Peer Review and Publication	6.6 MB	Page 15
20260220_paper_revision_highlighted.pdf	Additional File(s) for Peer Review but not for Publication	1.8 MB	Page 32
TOC.pdf	Graphical Abstract Image	851.0 KB	Page 38

Response to the Reviewers

Manuscript title: *Water in Solvate Ionic Liquids: Preserving Lithium Coordination While Enhancing Ionic Conductivity*
Journal: *ChemPhysChem*

We want to thank the editor and the reviewers for carefully reading our manuscript and for their constructive comments and questions. We have addressed all points raised and revised the manuscript accordingly. Reviewer comments are shown in **black**, our responses in **blue**, and changes made to the manuscript in **red**.

Response to Reviewer 1

Comment 1

The cathodic peak observed around -1.3 V is attributed to anion decomposition and the formation of a passivating interphase. While this assignment is reasonable by analogy with water-in-salt electrolytes, it relies largely on indirect evidence. If interphase formation is indeed dominant, the corresponding cathodic peak would diminish or disappear upon repeated cycling due to electrode passivation. Clarifying this behavior would strengthen the interpretation.

We want to thank the reviewer for his attentive feedback. As expected by the reviewer, the cathodic peaks disappear in measurements of a second CV scan due to the passivation of the electrodes. Recording the depicted CVs is only possible during the first scan. These data are not shown in the frame of this overview article and will be illustrated and discussed in detail in an upcoming publication. There, we will also consider that the electrodes are quicker passivated at higher temperatures and at higher water content.

Comment 2

The increase in cathodic current at potentials around -3 V is probably due to lithium deposition and/or electrolyte decomposition. However, oxidative current for lithium dissolution is likely absent in (1:1:1) systems. A more explicit discussion comparing the water-free (1:1:0) and water-containing (1:1:1) systems, particularly in terms of the absence or presence of anodic stripping features, would improve the discussion of the electrochemical properties.

We have not attributed the oxidation at 1 V vs Fc^+/Fc to the dissolution of lithium. Measurements at higher water content show that the oxidative peak is due to the inceptive oxidation of water. From a mixing ratio of [Li][NTf_2]:G3:H_2O = 1:1:4 onwards, the CV is dominated by water decomposition, and this determines the potential window (OER and HER). To discuss lithium deposition and anodic stripping, for our next publication, we will perform a surface analysis of the electrodes after oxidation/reduction employing XRD, XPS, and REM in connection with EDX to investigate the influence of water content on the ESWs. Furthermore, an

electrode passivation without oxidative re-dissolution of lithium is also supported by the fact that all electrodes had to be extensively polished after the CV scan.

Response to Reviewer 2

Comment 1

The authors attribute the wide ESW in the WISIL to the formation of a passivating SEI, which is in agreement with WIS literature. However, the current manuscript relies only on CV peaks to infer this. Is it possible to complement with additional surface analysis, for example, with XPS or SEM on the electrodes?

We want to thank the reviewer for the great feedback and for suggesting additional experiments to further validate our results. Unfortunately, we have not been able to perform such experiments yet. However, for an upcoming publication going into detail about the effect of water content on the ESWs, we intend to perform a surface analysis of the electrodes after oxidation/reduction employing XRD, XPS, and REM in connection with EDX. For now, we have softened the main text saying that the cathodic feature is indicative of SEI formation, and have added the suggestion of an additional surface analysis in the future:

> "Following Suo et al. and in analogy to classical WIS electrolytes, this cathodic feature is likely indicative of the formation of a solid electrolyte interphase (SEI). In WIS electrolytes, cathodic reduction of residual surface water molecules generates a locally alkaline environment near the electrode, which promotes anion reduction at potentials close to the hydrogen evolution reaction (HER). The resulting passivating SEI significantly extends the anodic limit of the ESW compared with conventional aqueous electrolytes. A very small cathodic feature at the same potential can also be discerned in the neat SIL, which may also originate from SEI formation. However, further surface analysis is required to confirm the composition of the passivating SEI."

Comment 2

The MD simulations underestimate experimental self-diffusion coefficients by a factor of 2. While the authors acknowledge this and focus on the trends, a brief discussion on whether polarizable force fields might resolve this quantitative discrepancy could be beneficial for future work.

We have added a sentence commenting on this to the manuscript:

> "While the chosen classical force fields reproduce the trends well, we note that polarizable models are increasingly available and offer promising improvements in accuracy, particularly for ionic liquids, and might therefore result in better quantitative agreement of simulation and experiment."

Comment 3

A minor anodic peak is attributed to ”trace amounts of free water”. It would be interesting to correlate this with the 1% water-water hydrogen bonding observed in simulations to see if the ”free water” can be more precisely defined.

At the moment, we cannot specify this more precisely with the current methodology at hand. However, we are grateful for the reviewer's comment and will consider it in future experiments and studies on this system.

Response to Reviewer 3

Comment 1

The authors attribute cathodic stability and specific CV features to SEI formation, yet they provide no surface characterization (e.g., XPS) to confirm the chemical composition of this interphase. This may also involve, e.g., the consumption of a slightly non-stoichiometric amount of water. In the discussion of Figure 5, ”we assign this cathodic feature primarily to the formation of a solid electrolyte interphase (SEI).” While this is a reasonable hypothesis based on the ”Water-in-Salt” literature (Suo et al.), the current manuscript does not present direct evidence of SEI formation (e.g., XPS, microscopy, or extended cycling data). Please soften the statement to indicate that this feature is likely indicative of SEI formation, or explicitly state that surface analysis is required to confirm the composition of the passivating layer.

We want to thank the reviewer for the positive review of our work and the valuable suggestion to prove our hypothesis with further investigations into the surface characteristics. Unfortunately, we have not been able to perform such experiments yet. However, for an upcoming publication going into detail about the effect of water content on the ESWs, we intend to perform a surface analysis of the electrodes after oxidation/reduction employing XRD, XPS, and REM in connection with EDX. For now, we have softened the main text saying that the cathodic feature is indicative of SEI formation, and have added the suggestion of an additional surface analysis in the future:

> “Following Suo et al. and in analogy to classical WIS electrolytes, this cathodic feature is likely indicative of the formation of a solid electrolyte interphase (SEI). In WIS electrolytes, cathodic reduction of residual surface water molecules generates a locally alkaline environment near the electrode, which promotes anion reduction at potentials close to the hydrogen evolution reaction (HER). The resulting passivating SEI significantly extends the anodic limit of the ESW compared with conventional aqueous electrolytes. A very small cathodic feature at the same potential can also be discerned in the neat SIL, which may also originate from SEI formation. However, further surface analysis is required to confirm the composition of the passivating SEI.”

Comment 2

While trends match, there is a notable quantitative difference between experimental and simulated diffusion/viscosity values, which the authors acknowledge but could discuss in slightly greater depth, noting force-field limitations. The manuscript notes that experimental self-diffusion coefficients are ≈2 times higher than MD results. While the Authors correctly identify that the ratios (D[Li] +/DG3) are consistent, the absolute deviation in viscosity may suggest that the force field is underestimating interactions or overestimating dynamics in the hydrated system. A brief sentence clarifying whether this is a known limitation of the specific G3 (TraPPE-UA) or water (TIP4P/2005) force field in ionic environments would strengthen the computational section.

We have added a sentence commenting on this to the manuscript:

> "While the chosen classical force fields reproduce the trends well, we note that polarizable models are increasingly available and offer promising improvements in accuracy, particularly for ionic liquids, and might therefore result in better quantitative agreement of simulation and experiment."

Comment 3

Concerns about water distribution: The claim that "only about 1% of the water molecules engage in [water-water] interactions" is a pivotal result distinguishing this system from WIS electrolytes. The visual representation in Figure 3c is excellent; however, a radial distribution function (RDF) of Water-Water interactions (O-O) and an additional table in the Supporting Information would be a helpful addition to demonstrate the lack of clustering quantitatively.

We have followed the suggestion by the reviewer and have added a figure to the SI showing the water-water O-O and H-O RDFs to the SI. Furthermore, we have added a sentence stating that the number of oxygen neighbors to a hydrogen atom can be calculated from the H-O RDFs, and thereby the percentage of water molecules being part of water-water clusters can be approximated to 1 % to 2 %. Unfortunately, the O-O RDFs cannot be used for computing the correct numbers of nearest neighbours since two water molecules that are coordinating the same lithium cation result in similar O-O distances as expected for the hydrogen bonds. Furthermore, this approach neglects the angle of the hydrogen bond O-H···O. Our cluster analysis was therefore performed with self-written code, considering a cutoff criterion for both the O-H distance and the O-H-O angle. The calculated percentages were 1.8 %. That is why the corresponding sentence in the main manuscript was altered. It now contains the correctly rounded number as well as a reference to the SI:

> "Consequently, only about 1.8 % of the water molecules engage in such interactions at both 303 K and 323 K (see SI for more details on computation). "

Comment 4

Figure 5 Caption/Text: The main text mentions that the ESW limits are determined at +/-0.1 mA*cm^2. Please explicitly state the current density cutoff criteria in the main text body for clarity, as this standard varies between publications.

We thank the reviewer for bringing up this important issue. We have added the cut-off criteria used for determining the ESWs also to the main text (additionally to the figure caption):

> "The broad ESW, determined at the current density limits of $\pm 0.1\,\mathrm{mA\,cm^{-2}}$, of the neat SIL of 5.2 V is only slightly reduced to 4.9 V in the WISIL."

We hope that the revisions satisfactorily address all comments. We would be happy to provide further clarification if needed.

WILEY-VCH

Water in Solvate Ionic Liquids: Preserving Lithium Coordination While Enhancing Ionic Conductivity

Jule Kristin Philipp,*[a] Dietmar Paschek,*[a] Lennart Kruse,[a] Annette-Enrica Surkus,[b] Bennet Austrup,[c] Monika Schönhoff,[c] Ralf Ludwig,*[a,b,d]

This work investigates how the concepts of solvate ionic liquids (SILs) and water-in-salt (WIS) electrolytes can be combined to create hybrid electrolyte systems. We examine the neat SIL [Li(G3)][NTf_2], composed of a solvate cation and an anion, as well as its water-modified analogue containing an equimolar amount of added water, using both molecular dynamics (MD) simulations and experiments. Introducing water markedly reduces the high viscosity of the neat SIL while substantially enhancing ionic conductivity. Structurally, each cationic complex incorporates on average a single water molecule, resulting in highly dispersed water and the absence of extended water networks. We refer to such systems as *water-in-solvate-ionic-liquid* (WISIL) electrolytes. Owing to the strongly coordination-dominated lithium environment, the WISIL retains a wide electrochemical stability window, decreasing only slightly from over 5 V in the neat SIL to 4.9 V at ambient conditions.

Solvate ionic liquids (SILs) represent a distinct class of liquid electrolytes composed of a salt and a molecular solvent capable of forming a stable chelate complex depending on the mixing ratio.[1–4] This unique coordination gives rise to physicochemical properties comparable to those of conventional ionic liquids (ILs).[5–7] In addition, SILs exhibit remarkably high thermal stability, further broadening their applicability in electrochemical systems.[4] By tailoring the composition of a SIL, these properties can be finely tuned, making them promising candidates for safer and more environmentally friendly battery electrolytes.[6–9]

A well-studied class of SILs are equimolar mixtures of lithium bis(trifluoromethanesulfonyl)imide ([Li][NTf_2]) with glyme solvents (H-(CH_2-O-CH_2)$_n$-H), such as triglyme (G3, $n = 4$).[6] Their distinct coordination chemistry leads to well-defined complex cations, e.g., $[Li(G3)]^+$, which exhibit high thermal and electrochemical stability. Nevertheless, the high viscosities and correspondingly low ionic conductivities of these systems limit their practical applicability.[4–6]

Previous studies have shown that dilution with molecular solvents can substantially enhance the ionic conductivity of SILs by lowering viscosity.[6,10–12] However, the nature of the additive plays a crucial role. Nonpolar solvents primarily act as viscosity modifiers without significantly destabilizing the cation–glyme complexes. In contrast to this, Ueno et al. demonstrated that highly polar solvents such as water can interfere with the $[Li]^+$ solvation environment by competing with glyme ligands for coordination.[10,12,13]

Despite these challenges, aqueous electrolytes remain attractive due to their inherent safety and sustainability. However, their narrow electrochemical stability window (ESW) limits their applicability in systems operating at the electrochemical potentials of conventional Li-ion battery electrodes.[10,14] To overcome these limitations, a new class of electrolytes, water-in-salt (WIS) systems, has recently emerged. In these highly concentrated aqueous electrolytes, where the salt content exceeds that of water in both mass and volume, lithium cations remain closely associated with their counterions rather than being fully hydrated. Such water-rich yet ion-dense systems exhibit remarkably broad ESWs of up to ≈ 3 V at room temperature, compared to 1.23 V for conventional aqueous electrolytes, due to the formation of stable interphases that suppress water reduction.[15–20]

In this work, we aim to combine the advantages of SIL and WIS electrolytes by introducing an equimolar amount of water into the SIL [Li(G3)][NTf_2]. Specifically, we address three key questions: (1) How does the addition of water influence the transport and electrochemical properties of the SIL? (2) How does water affect the local solvation structure of lithium cations? and (3) What specific role does water play at such low concentrations in determining the overall physicochemical behavior of the electrolyte?

To address these questions, we investigated the neat SIL ([Li][NTf_2]:G3:H_2O = 1:1:0) and its water-modified analogue containing an equimolar amount of water ([Li][NTf_2]: G3:H_2O = 1:1:1) using both experiments and molecular dynamics (MD) simulations at 303 K and 323 K. Two MD setups were employed: initial simulations in a cubic box, followed by multi-microsecond orthorhombic simulations using the OrthoBoXY approach by Busch and Paschek to determine system-size independent self-diffusion coefficients and viscosities.[22,23] The simulations were complemented by experimental measurements of transport properties, including self-diffusion coefficients from pulsed-field gradient NMR[24] and electrochemical behaviour from cyclic voltam-

[a] J. K. Philipp*, Dr. D. Paschek*, L. Kruse, Prof. R. Ludwig*
Institut für Chemie, Physikalische und Theoretische Chemie, Universität Rostock, Albert-Einstein-Straße 27, D-18059 Rostock, Germany
E-mail: jule.philipp@uni-rostock.de
dietmar.paschek@uni-rostock.de
ralf.ludwig@uni-rostock.de

[b] Dr. A.-E. Surkus, Prof. R. Ludwig*
Leibniz-Institut für Katalyse (LIKAT) an der Universtät Rostock, Albert-Einstein-Straße 29a, D-18059 Rostock, Germany

[c] B. Austrup, Prof. M. Schönhoff
Institute of Physical Chemistry, University of Münster, Corrensstr. 28/30, D-48149 Münster, Germany

[d] Prof. R. Ludwig*
Department LL&M, Universität Rostock, Albert-Einstein-Straße 25, D-18059 Rostock, Germany

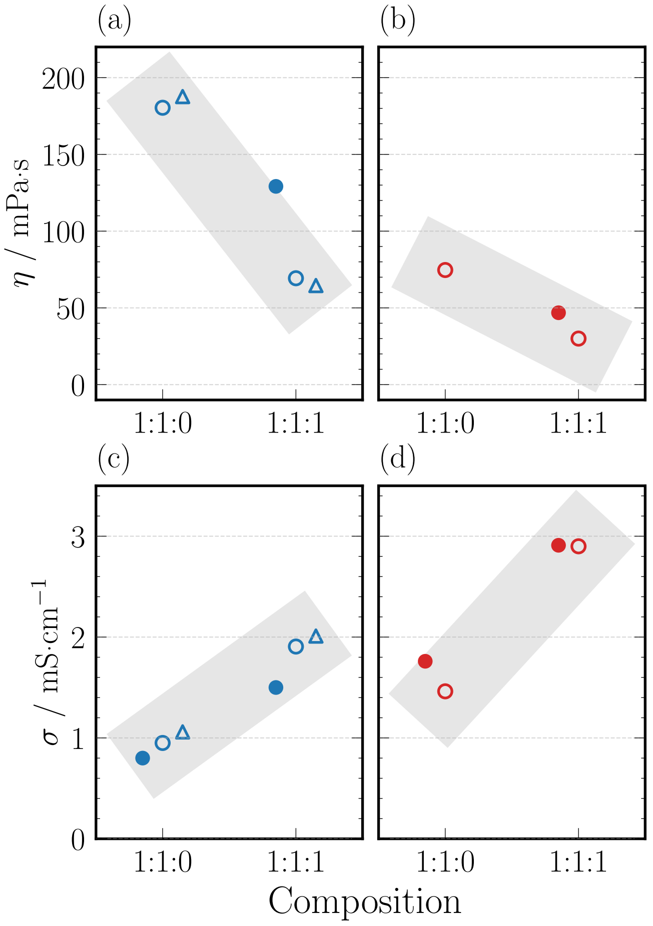

Figure 1. Shear viscosity η (a,b) and ionic conductivity σ (c,d) obtained from experiment (open circles) as well as MD simulations (filled circles) at 303 K (blue) and 323 K (red). Mixture compositions 1:1:0 and 1:1:1 correspond to [Li][NTf_2]:G3:H_2O. Experimental reference data at 303 K (open triangles) are taken from refs.[10,21]. Grey bars serve as visual guides to highlight trends; individual data points are horizontally offset for clarity.

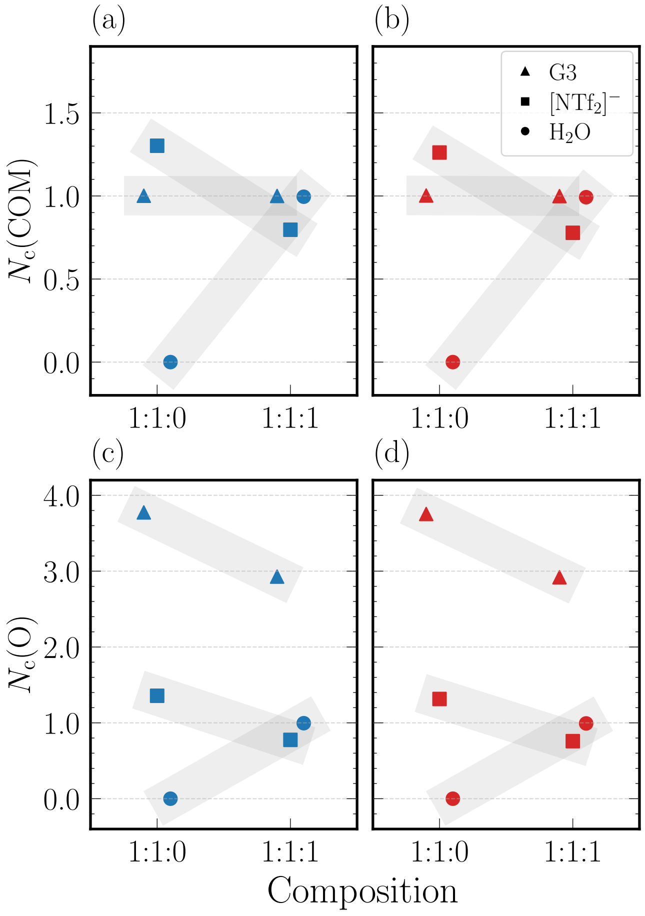

Figure 2. Coordination numbers of $[Li]^+$ with G3 (triangles), $[NTf_2]^-$ (squares), and H_2O (circles) from MD simulations at 303 K (blue) and 323 K (red). N_c(COM) (a,b) denotes the average number of molecules coordinated to $[Li]^+$ based on center-of-mass distances, while oxygen coordination numbers (c,d) represent atomic-level contributions. Mixture compositions 1:1:0 and 1:1:1 correspond to [Li][NTf_2]:G3:H_2O. Grey bars serve as visual guides to highlight trends; individual data points are horizontally offset for clarity.

metry. Detailed descriptions of all experimental procedures and simulation protocols are provided in the Supporting Information.

Experimental densities are well reproduced by the MD simulations for both compositions and temperatures (see SI), providing confidence in the employed molecular model and the microscopic interpretation developed in this study.

Figure 1 summarises the experimental and simulated shear viscosities and ionic conductivities of both systems at the two investigated temperatures. The addition of water has a pronounced effect on the shear viscosity. The neat SIL exhibits a very high viscosity of 180 mPa s at 303 K, which decreases by more than half upon heating to 323 K. Introducing water substantially lowers the viscosity to 69 mPa s at 303 K and to 30 mPa s at 323 K. Viscosities obtained from MD simulations are of the same order of magnitude as the experimental values. However, the high viscosities necessitate long simulation trajectories to achieve acceptable statistical accuracy. Although multi-microsecond simulations still lead to noticeable deviations from experiment, the simulations reproduce the experimental trends well.

The ionic conductivity follows the opposite trend. The neat SIL shows a low conductivity of 0.95 mS cm^{-1} at 303 K, nearly an order of magnitude smaller than that of conventional electrolytes.[25] The water-modified system, however, exhibits an approximately twofold increase in conductivity. At 323 K, the conductivity rises from 1.46 mS cm^{-1} to 2.90 mS cm^{-1}, in near-quantitative agreement with MD simulations. At 303 K, simulations slightly underestimate the conductivity, likely due to the limited ion mobility and associated statistical uncertainty in this highly viscous regime.

Overall, the experimental and simulated results are in very good agreement across the measured properties. This consistency demonstrates that the molecular models accurately capture the macroscopic behavior of these complex electrolytes and supports the reliability of the simulated structural motifs in representing the real liquid systems.

Figure 2 provides insights into the local coordination environment of the lithium cations at both investigated temperatures. Overall, both systems exhibit similar lithium coordination numbers within the studied temperature range, as derived from the data in Table S5. As shown in a previous study, the characteristic coordination motif of the neat SIL remains largely unchanged up to 483 K.[4] In the binary, water-free system, each lithium cation is on average

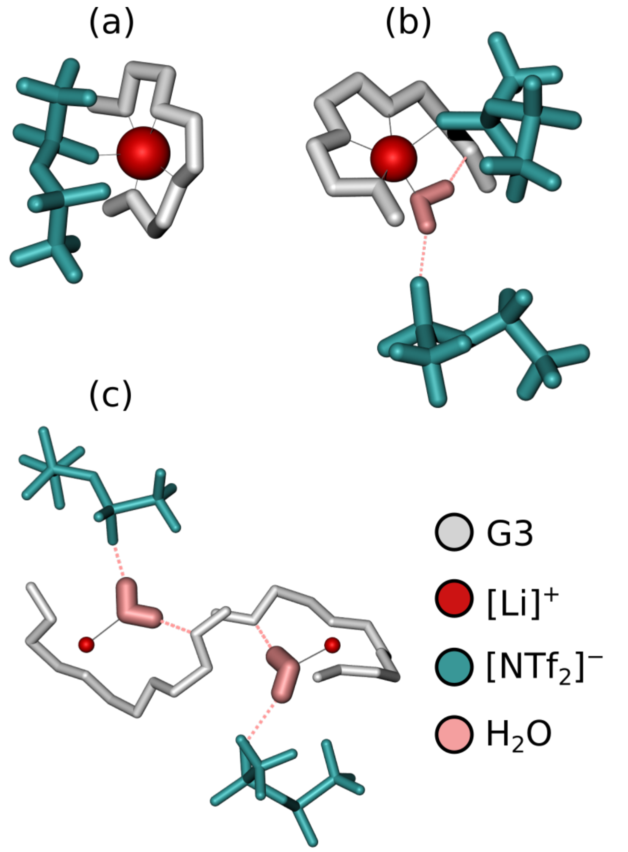

Figure 3. Typical lithium environment for the mixtures with the [Li][NTf_2]:G3:H_2O composition of (a) 1:1:0 (neat SIL) and (b) 1:1:1, while (c) highlights the spatial separation of water molecules due to the incorporation in complex cations in the water-modified SIL. All snapshots are taken from the MD simulations at 303 K. [NTf_2]$^-$ anions, [Li]$^+$ cations, G3 molecules, and H_2O molecules are depicted in cyan, red, grey, and pink, respectively. Coordinative bonds are illustrated by black, solid lines, while HBs are shown as pink, dashed lines.

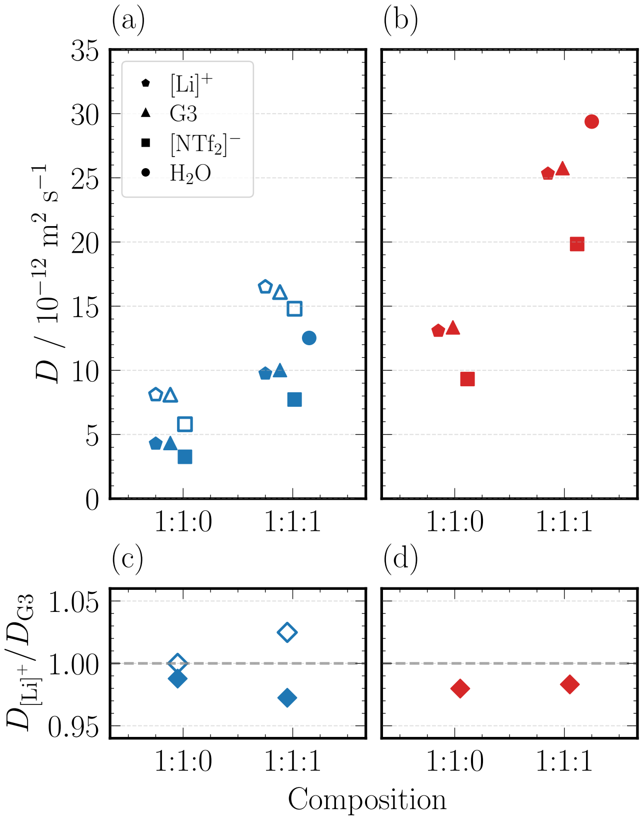

Figure 4. Self-diffusion coefficients of [Li]$^+$ (pentagons), G3 (triangles), [NTf_2]$^-$ (squares), and H_2O (circles) from MD simulations (filled symbols) at (a) 303 K (blue) and (b) 323 K (red). At 303 K, experimentally obtained values are given as open symbols. (c) and (d) Ratio of self-diffusion coefficients of [Li]$^+$ and G3 obtained from MD simulations (closed symbols) and experiment (open symbols) at 303 K (blue diamonds) and 323 K (red diamonds), respectively. Mixture ratios of 1:1:0 and 1:1:1 correspond to [Li][NTf_2]:G3:H_2O, respectively. Individual data points are horizontally offset for clarity.

coordinated by a single triglyme molecule, whose four oxygen atoms form a stable chelate complex. The coordination sphere is further complemented by anions, with one or two [NTf_2]$^-$ anions contributing one oxygen atom each. A representative snapshot of the most abundant structural motif in the neat SIL is shown in Figure 3a.

Upon addition of an equimolar amount of water, the coordination environment of lithium undergoes subtle but distinct changes. Most lithium cations remain associated with a single triglyme molecule; however, in the majority of cases, only three of its oxygen atoms now directly participate in coordination. The fourth site—most likely one of the two terminal oxygen atoms of the G3 molecule (see Table S8 and Figure S4)—is replaced by a water molecule. This water molecule enters the first coordination shell and simultaneously forms hydrogen bonds (HBs) with both the displaced triglyme oxygen and a nearby anion (Figure 3b). Furthermore, the average number of anions in the first solvation shell decreases to below one, with some anions partially replaced by water. As a result, the overall lithium–oxygen coordination number decreases from 5.1 to 4.7. Additional details on the distribution of structural motifs surrounding [Li]$^+$ are provided in the Supporting Information.

The stability of such complex cations can be assessed by comparing the self-diffusion coefficients of the lithium cation and the coordinating glyme. The ratio $D_{[\mathrm{Li}]^+}/D_{\mathrm{G3}}$ reflects whether lithium and G3 diffuse together, with values near unity indicating stable cationic complexes.[5,12] As shown in Figure 4c/d, this ratio remains close to unity for both the neat and water-modified SIL at 303 K and 323 K in experiment and simulation. The underlying self-diffusion coefficients are demonstrated in Figure 4a for 303 K and in Figure 4b for 323 K. Although experimentally measured self-diffusion coefficients are generally enhanced by a factor of ≈ 2 compared to MD simulation results, they increase with temperature and are consistently higher in the water-containing system, reflecting the reduced viscosity. Yet, the relative mobilities of [Li]$^+$ and G3 remain similar. This finding demonstrates that adding an equimolar amount of water does not significantly disrupt the integrity of the complex cations. While the chosen classical force fields reproduce the trends well, we note that polarizable models are increasingly available and offer promising improvements in accuracy, particularly for ionic liquids[26], and might therefore result in better quantitative agreement of simulation and experiment.

The incorporation of water into the lithium coordination sphere results in the effective spatial separation and dispersion of individual water molecules throughout the system.

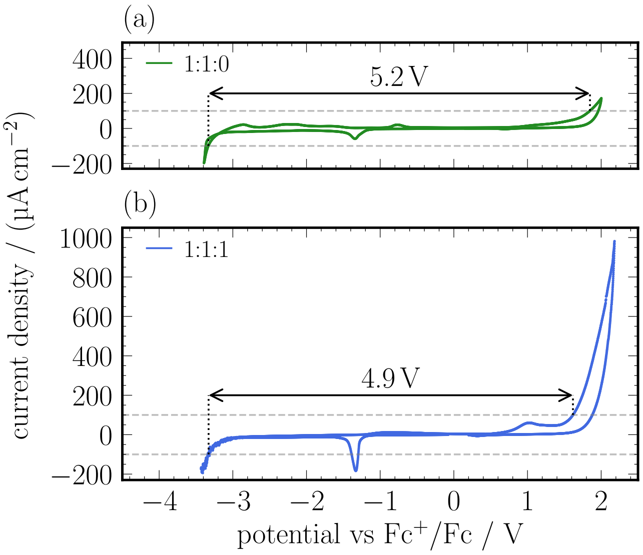

Figure 5. CV curves of (a) the neat SIL (green, measured at 303 K) as well as (b) with an equimolar amount of water added (blue, measured at 296 K). The respective ESWs are denoted above each CV curve. Grey dashed lines mark the limits for determining the respective ESWs at $-0.1\,\mathrm{mA\,cm^{-2}}$ and $0.1\,\mathrm{mA\,cm^{-2}}$. Mixture ratios of 1:1:0 and 1:1:1 correspond to [Li][NTf_2]:G3:H_2O, respectively.

This behaviour is illustrated in Figure 3c, where neighbouring complex cations each contain a single, internally coordinated water molecule that is sterically hindered from forming water–water HBs. Consequently, only about below 1.8 % of the water molecules engage in such interactions at both 303 K and 323 K (see SI for more details on computation).

Because the characteristic structural motif of the 1:1:1 system combines features of both disrupted water networks typical of WIS electrolytes and the stable chelate complexes characteristic of SILs, we introduce the term *water-in-solvate-ionic-liquid* (WISIL) electrolyte for this water-modified SIL.

At both temperatures, more than 99 % of all triglyme and water molecules participate in lithium coordination. This coordination-dominated environment is advantageous in SILs, as it minimises the amount of free solvent and thereby reduces solvent degradation at the electrodes.[7] The absence of bulk-like water clusters and the extremely low fraction of uncoordinated solvent molecules suggest an ESW wider than that of conventional aqueous electrolytes and comparable to WIS systems.

Cyclic voltammetry (CV) measurements (see Figure 5) confirm this: the broad ESW, determined at the current density limits of $\pm 0.1\,\mathrm{mA\,cm^{-2}}$, of the neat SIL of 5.2 V is only slightly reduced to 4.9 V in the WISIL. The 1:1:1 WISIL, however, shows a minor anodic peak at 1.03 V to 1.07 V vs Fc^+/Fc attributed to the oxidation of trace amounts of free water at the anode. In addition, a distinct cathodic peak at −1.33 V vs Fc^+/Fc emerges. Following Suo et al. and in analogy to classical WIS electrolytes, this cathodic feature is likely indicative of the formation of a solid electrolyte interphase (SEI).[15,17] In WIS electrolytes, cathodic reduction of residual surface water molecules generates a locally alkaline environment near the electrode, which promotes anion reduction at potentials close to the hydrogen evolution reaction (HER). The resulting passivating SEI significantly extends the anodic limit of the ESW compared with conventional aqueous electrolytes. A very small cathodic feature at the same potential can also be discerned in the neat SIL, which may also originate from SEI formation. However, further surface analysis is required to confirm the composition of the passivating SEI.

To conclude, experimental data and MD simulations consistently show that adding an equimolar amount of water to the neat SIL [Li(G3)][NTf_2] improves the transport properties of the modified SIL by reducing viscosity and thereby doubling ionic conductivity. Structurally, lithium remains coordinated by one G3 molecule, with a single water molecule substituting one donor site and forming hydrogen bonds to the G3 ligand and nearby anions. Despite this modification, the self-diffusion coefficient ratio of $[Li]^+$ and G3 remains close to unity, confirming the persistence of long-lived complex cations. The dispersed, non-associating water molecules enhance transport without disrupting the SIL structure, acting as a molecular lubricant, while maintaining a broad ESW, probably due to the formation of a passivating SEI. Previous studies have shown, however, that the beneficial effects are lost when larger amounts of water are introduced, leading to the breakdown of the complex structure.[10] These insights highlight a narrow but valuable compositional window for optimising SIL-based electrolytes and motivate further studies toward fine-tuning solvation structure and ion mobility for next-generation electrolytes.

Acknowledgements

JKP, DP, and RL thank A. Wilhelms for measuring densities, viscosities, and ionic conductivities. This work has been supported by Deutsche Forschungsgemeinschaft (DFG) with Research Grants LU-506/17-1 (project no. 470038970) and LU-506/18-1 (project no. 517661181).

Conflict of Interest

The authors declare no conflict of interest.

Data Availability Statement

The code of GROMACS is freely available. Input parameter and topology files for the MD simulations can be downloaded from GitHub via
https://github.com/Paschek-Lab/WISIL-G3/

Keywords: Solvate Ionic Liquid • Water-in-Salt Electrolyte • Molecular Dynamics Simulations • Structure • Cluster Formation

References

[1] T. Tamura, K. Yoshida, T. Hachida, M. Tsuchiya, M. Nakamura, Y. Kazue, N. Tachikawa, K. Dokko, M. Watanabe, Chem. Lett. **2010**, 39, 753.
[2] T. Tamura, T. Hachida, K. Yoshida, N. Tachikawa, K. Dokko, M. Watanabe, J. Power Sources **2010**, 195, 6095.
[3] J. K. Philipp, K. Fumino, A. Appelhagen, D. Paschek, R. Ludwig, ChemPhysChem **2025**, 26, e202400991.
[4] J. K. Philipp, L. Kruse, D. Paschek, R. Ludwig, J. Phys. Chem. B **2025**, 129, 5561.
[5] K. Ueno, Electrochemistry **2016**, 84, 674.
[6] K. Ueno, J. Murai, H. Moon, K. Dokko, M. Watanabe, J. Electrochem. Soc. **2016**, 164, A6088.
[7] T. Harte, B. Dharmasiri, Ž. Simon, D. J. Hayne, D. J. Eyckens, L. C. Henderson, J. Mater. Chem. A **2025**, 13, 12746.
[8] D. J. Eyckens, L. C. Henderson, Front. Chem. **2019**, 7, 263.
[9] M. Watanabe, Glyme-based Solvate Ionic Liquids and Their Electrolyte Properties, in K. Yamamoto, H. Nishihara (Editors), Functional Macromolecular Complexes, pages 271–295, Royal Society of Chemistry **2024**.
[10] K. Ueno, J. Murai, K. Ikeda, S. Tsuzuki, M. Tsuchiya, R. Tatara, T. Mandai, Y. Umebayashi, K. Dokko, M. Watanabe, J. Phys. Chem. C **2016**, 120, 15792.
[11] H. Shobukawa, K. Shigenobu, S. Terada, S. Kondou, K. Ueno, K. Dokko, M. Watanabe, Electrochim. Acta **2020**, 353, 136559.
[12] K. Shigenobu, T. Sudoh, J. Murai, K. Dokko, M. Watanabe, K. Ueno, Chem. Rec. **2023**, 23, e202200301.
[13] T. Sudoh, K. Shigenobu, K. Dokko, M. Watanabe, K. Ueno, Phys. Chem. Chem. Phys. **2022**, 24, 14269.
[14] T. F. Burton, R. Jommongkol, Y. Zhu, S. Deebansok, K. Chitbankluai, J. Deng, O. Fontaine, Curr. Opin. Electrochem. **2022**, 35, 101070.
[15] L. Suo, O. Borodin, T. Gao, M. Olguin, J. Ho, X. Fan, C. Luo, C. Wang, K. Xu, Science **2015**, 350, 938.
[16] L. Suo, O. Borodin, W. Sun, X. Fan, C. Yang, F. Wang, T. Gao, Z. Ma, M. Schroeder, A. von Cresce, S. M. Russell, M. Armand, A. Angell, K. Xu, C. Wang, Angew. Chem., Int. Ed. **2016**, 55, 7136.
[17] N. Dubouis, P. Lemaire, B. Mirvaux, E. Salager, M. Deschamps, A. Grimaud, Energy Environ. Sci. **2018**, 11, 3491.
[18] M. Amiri, D. Bélanger, ChemSusChem **2021**, 14, 2487.
[19] A. Tot, L. Zhang, E. J. Berg, P. H. Svensson, L. Kloo, Sci. Rep. **2023**, 13, 2154.
[20] D. Dong, C.-X. Zhao, X. Zhang, C. Wang, Adv. Mater. **2025**, 37, 2418700.
[21] C. Zhang, K. Ueno, A. Yamazaki, K. Yoshida, H. Moon, T. Mandai, Y. Umebayashi, K. Dokko, M. Watanabe, J. Phys. Chem. B **2014**, 118, 5144.
[22] J. Busch, D. Paschek, J. Phys. Chem. B **2023**, 127, 7983.
[23] J. Busch, D. Paschek, J. Phys. Chem. B **2024**, 128, 1040.
[24] M. Holz, S. R. Heil, A. Sacco, Phys. Chem. Chem. Phys. **2000**, 2, 4740.
[25] K. Xu, Chem. Rev. **2004**, 104, 4303.
[26] D. Bedrov, J.-P. Piquemal, O. Borodin, A. D. J. MacKerell, B. Roux, C. Schröder, Chem. Rev. **2019**, 119, 7940.

Entry for the Table of Contents

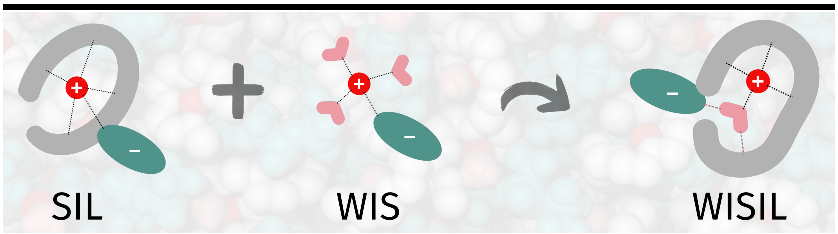

By adding a controlled amount of water to a solvate ionic liquid (SIL), this concept can be bridged with water-in-salt (WIS) electrolytes. Water is inserted into the characteristic cationic complex, leading to *water-in-solvate-ionic-liquid* (WISIL) electrolytes.

Supporting Information:

Water in Solvate Ionic Liquids: Preserving Lithium Coordination While Enhancing Ionic Conductivity

Jule Kristin Philipp[1,⋆], Dietmar Paschek[1,⋆], Lennart Kruse[1], Annette-Enrica Surkus[2], Bennet Austrup[3], Monika Schönhoff[3], and Ralf Ludwig[1,2,4,⋆]

[1] *Institut für Chemie, Physikalische und Theoretische Chemie, Universität Rostock, Albert-Einstein-Straße 27, D-18059 Rostock, Germany*
[2] *Leibniz-Institut für Katalyse (LIKAT) an der Universität Rostock, Albert-Einstein-Straße 29a, D-18059 Rostock, Germany*
[3] *Institute of Physical Chemistry, University of Münster, Corrensstr. 28/30, D-48149 Münster, Germany*
[4] *Department LL&M, Universität Rostock, Albert-Einstein-Straße 25, D-18059 Rostock, Germany*

⋆**E-mail:** jule.philipp@uni-rostock.de, dietmar.paschek@uni-rostock.de, ralf.ludwig@uni-rostock.de

Contents

S1 Experimental Procedures & Results **S3**
S1.1 Preparation of Water-Modified Solvate Ionic Liquids . . . S3
S1.2 Density . . . S3
S1.3 Shear Viscosity . . . S3
S1.4 Ionic conductivity . . . S3
S1.5 Self-Diffusion Coefficients . . . S4
S1.6 Electrochemical Characterisation . . . S6

S2 MD Simulation Protocols & Results **S7**
S2.1 Simulation Setup . . . S7
S2.2 OrthoBoXY Simulations & Viscosities . . . S8
S2.3 Radial Distribution Functions . . . S10
S2.4 Self-Diffusion Coefficients . . . S10
S2.5 Ionic Conductivity . . . S11

S2.6 Coordination Numbers S12
S2.7 Water-water cluster analysis S13
S2.8 Structural Motifs S14

S1 Experimental Procedures & Results

S1.1 Preparation of Water-Modified Solvate Ionic Liquids

Lithium bis(trifluoromethylsulfonyl)imide ([Li][NTf_2], 99.99 %, anhydrous) and triethylenglycoldimethylether (triglyme/G3, 99 %) were purchased from Sigma-Aldrich. The triglyme was dried over freshly activated molecular sieves (3 Å and 4 Å) and the water content was confirmed to be below 10 ppm by Karl-Fischer titration. In a glovebox, the glyme (10.95 g) was added to a Schlenk tube. Then, the [Li][NTf_2] (17.63 g) was added, and the solution was stirred for two days at room temperature until everything was dissolved and homogeneous. Ultrapure water was added outside the glovebox with a pipette, while purging the Schlenk tube with inert gas. Again, the solution was stirred until it became homogeneous.

S1.2 Density

The experimental densities (see Table S1) were obtained using an Anton Paar DMA 5000 M density meter with an accuracy of $\delta\rho = \pm 0.007\,\mathrm{kg\,m^{-3}}$ and $\delta T = \pm 0.01\,\mathrm{K}$ (according to Anton Paar Technical Manual).

S1.3 Shear Viscosity

The experimental viscosities reported in the main manuscript were obtained using a rolling ball viscometer Lovis 2000 M/ME from Anton Paar, and are presented in Table S1 with accuracy of $\delta\eta/\eta = \pm 0.5\,\%$ and $\delta T = \pm 0.02\,\mathrm{K}$ (according to Anton Paar Technical Manual).

S1.4 Ionic conductivity

The ionic conductivities in Table S1 were determined using a Fisher Scientific Orion Star3 conductivity meter. This device has a measurement accuracy of 0.5 % of the measured value ±1 digit for values greater than 3 µS.

Table S1: Experimentally obtained physicochemical properties of SIL [Li(G3)][NTf_2] with and without an equimolar amount of water added at 303 K and 323 K.

T	303 K		323 K	
[Li][NTf_2]:G3:H_2O	1:1:0	1:1:1	1:1:0	1:1:1
ρ / $\mathrm{kg\,m^{-3}}$	1425.972	1399.826	1405.735	1380.007
η / mPa s	180.4	69.31	74.67	30.00
σ / $\mathrm{mS\,cm^{-1}}$	0.950	1.906	1.462	2.90

S1.5 Self-Diffusion Coefficients

Materials Lithium bis(trifluoromethylsulfonyl)imide ([Li][NTf_2]; Sigma-Aldrich, $\geq 99\,\%$, dried at 10 hpa and 100 °C overnight) was mixed with triethylene glycol dimethyl ether (G3, Sigma Aldrich, $\geq$ 99 %, dried with molecular sieve) at a molar glyme/salt ratio of 1:1. Water was added in the same ratio.

NMR Experiment All NMR measurements were conducted on an AVANCE Neo 400 MHz NMR spectrometer (Bruker, Rheinstetten, Germany) using a gradient probe head ("DiffBB", Bruker) with a maximum magnetic field gradient strength of 17 T/m. The ^{1}H, ^{7}Li, and ^{19}F experiments were conducted at 303 K on liquid samples in standard 5 mm NMR tubes.

PFG-NMR Diffusion For diffusion measurements, a stimulated echo (STE) pulse sequence was used where the gradient pulse duration δ (up to 1.5 ms) was adjusted for each experiment, and the observation time Δ was set to 100 ms. A sequence of spectra was recorded while systematically varying the gradient strength g (up to 17 T/m). For each experiment, g was increased stepwise until the corresponding echo signal had decayed completely. The sample temperature was controlled through the temperature setting of the gradient cooling unit, operated without airflow. Calibration of the actual sample temperature was performed using a reference tube equipped with a PT100 thermocouple. The diffusion coefficient D was determined from an exponential fit of the g-dependent intensity decay according to the Stejskal-Tanner equation (eq. 1) with γ representing the gyromagnetic ratio.[1]

$$I = I_0 \exp(D\gamma^2\delta^2 g^2(\Delta - \frac{\delta}{3})) = I_0 \exp(-kD) \tag{1}$$

In the case of the ^{1}H spectra of the 1:1:1 sample, I was obtained by integration over the spectral region of those G3 signals, which did not overlap with the H_2O signal. Due to the overlap and its comparatively low intensity, the water resonance was not evaluated separately.

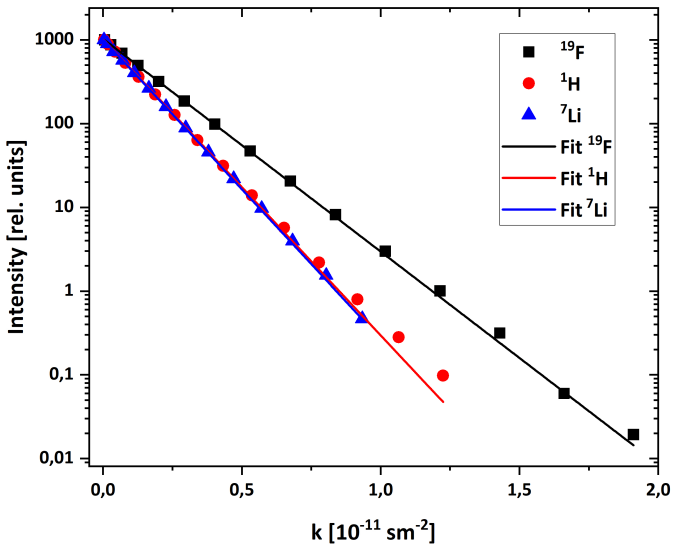

Figure S1: Echo decay curves of the PFG-stimulated echo sequence with gradient strengths g up to 17 T/m for the ^{1}H, ^{7}Li, and ^{19}F nuclei in [Li][NTf$_2$]:G3:H$_2$O = 1:1:0 at 303 K, representing G3, lithium and anion diffusion, respectively.

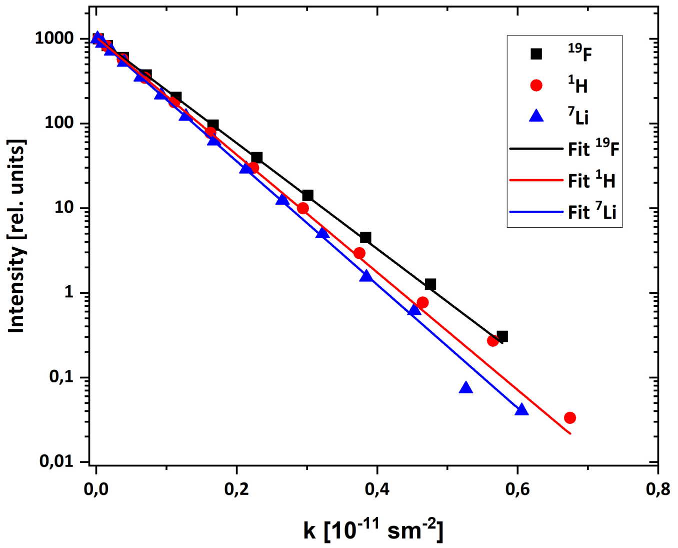

Figure S2: Echo decay curves of the PFG-stimulated echo sequence with gradient strengths g up to 17 T/m for the ^{1}H, ^{7}Li, and ^{19}F nuclei in [Li][NTf$_2$]:G3:H$_2$O = 1:1:1 at 303 K, representing G3, lithium and anion diffusion, respectively.

Table S2: Experimentally obtained self-diffusion coefficients of SIL [Li(G3)][NTf_2] with and without an equimolar amount of water added at 303 K.

	D / 10^{-11} $m^2\,s^{-1}$	
[Li][NTf_2]:G3:H_2O	1:1:0	1:1:1
^{19}F ([NTf_2]$^-$)	$0,58 \pm 0,03$	$1.48 \pm 0,06$
^{1}H (G3)	$0,81 \pm 0,04$	$1.61 \pm 0,07$
^{7}Li ([Li]$^+$)	$0,81 \pm 0,04$	$1.65 \pm 0,07$

S1.6 Electrochemical Characterisation

All investigations were performed at a thermostatically controlled temperature under an argon atmosphere using an Autolab PGSTAT 128N (Metrohm). The working electrode (WE), counter electrode (CE), and reference electrode (RE) were a platinum disk electrode (d = 3 mm), a platinum rod, and a silver wire, respectively (WE and CE: Metrohm). All reported potentials were corrected for the IR drop and converted using the ferrocenium/ferrocene internal reference system. CV scans were performed once at a scan rate of 1 mV s^{-1}, starting in the cathodic direction. Uncompensated resistance (R) was determined by electrochemical impedance spectroscopy (EIS) at open-circuit potential (OCP) with an alternating current perturbation of 5 mV and a frequency range of 20 kHz to 200 Hz.

S2 MD Simulation Protocols & Results

S2.1 Simulation Setup

We have performed MD simulations of the neat SIL ([Li][NTf_2]:G3:H_2O = 1:1:0) and its water-modified analogue containing an equimolar amount of water ([Li][NTf_2]:G3:H_2O = 1:1:1) at 303 K and 323 K. For the description of the lithium ions, we used the forcefield for monovalent alkali cations by Joung and Cheatham.[2] Besides, the NGOLP forcefield was applied for the $[NTf_2]^-$ anions.[3,4] As a force field for G3, we have relied on the modified TraPPE-UA force field of Fischer et al.[5–7], which has recently been compared favorably to various other forcefields.[8] As for water, we have relied on the TIP4P/2005 forcefield.[9]

All MD simulations were performed using GROMACS 2019.6[10–12]. The simulations were carried out under NpT conditions at a pressure of 1 bar. The binary system consisted of 540 [Li][NTf_2] ion pairs (IPs) and 540 G3 molecules in a cubic simulation box. For the ternary system, 540 water molecules were additionally added. All initial simulation box configurations were generated employing the software package Packmol.[13] Each system was first equilibrated for 20 ns at 383 K. The final configuration from equilibration served as the initial configuration for the 200 ns production run at the same temperature. The final configuration of the production run was then used as the start configuration for the next lower temperature. Temperatures were chosen in a 20 K interval until reaching 303 K. All simulations used a 2 fs integration time step. The temperature of the simulated systems was controlled by employing the Nosé–Hoover thermostat[14–16] with a coupling time $\tau_T = 1.0$ ps. Here, we have followed the recommendations given by Basconi and Shirts[17], who found that the application of the Nosé-Hoover thermostat yields reliable dynamical properties that are statistically indistinguishable from those of the microcanonical (NVE) ensemble, given that it is applied globally. Additionally, the Parrinello-Rahman barostat[18] was applied with a coupling time $\tau_p = 2.0$ ps. The electrostatic and Lennard-Jones interactions were treated by the smooth particle mesh Ewald summation.[19,20] The Ewald convergence parameter was set to a relative accuracy of the Ewald sum of 1×10^{-5} for the Coulomb interactions and 1×10^{-3} for the Lennard-Jones interactions. All bond lengths were kept fixed during the simulation run, and the distance constraints were solved utilizing the LINCS procedure.[21] Snapshots of the simulation boxes at 303 K obtained by employing these parameters are depicted for the neat SIL and the water-modified system in Figure S3.

All trajectory data were stored in a wrapped form, with coordinates of the molecules folded back into the central MD box. Hence, for the computation of the mean squared displacements (MSDs) of the molecules, the trajectory data needed to be properly unwrapped. To unfold our NpT simulation data, we relied on a procedure similar to the toroidal-view-preserving scheme discussed in ref.[22] to avoid artifacts that could lead to unphysically large self-diffusion coefficients[23], possibly also affecting all related transport properties.

The computation of the properties from MD simulations was performed using our home-built software package MDorado[24], which is available via GitHub.

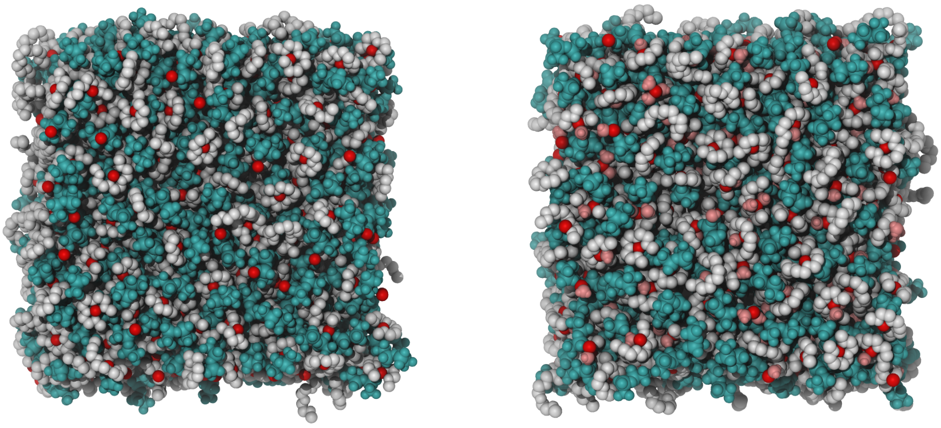

(a) (b)

Figure S3: Snapshots of the cubic MD simulation boxes at 303 K for (a) the neat $[Li(G3)][NTf_2]$ SIL and (b) its water-modified analogue with an equimolar amount of water molecules added. G3 molecules are coloured gray, $[Li]^+$ cations red, $[NTf_2]^-$ anions cyan, and H_2O molecules pink.

S2.2 OrthoBoXY Simulations & Viscosities

For calculating system-size independent self-diffusion coefficients D_0 and viscosities η, the OrthoBoXY method was employed.[25–27]

By choosing an orthorhombic box geometry with "magic" box length ratios of $L_z/L_x = L_z/L_y \approx 2.7933596497$, the shear viscosity can be obtained as

$$\eta = \frac{k_\mathrm{B} T \zeta_{zz}}{6\pi L_z (D_0 - D_{\mathrm{PBC},zz})} \tag{2}$$

with the Madelung constant analogue in the z-direction of $\zeta_{zz} \approx 8.1711245653$ and L_z being the box length in z-direction.[27]

For a multi-component mixture of P components, the overall shear viscosity is computed as a weighted component average according to

$$\eta = \frac{k_\mathrm{B} T \zeta_{zz}}{6\pi L_z} \cdot \sum_{p=1}^{P} \frac{x_p}{D_{0,p} - D_{\mathrm{PBC},zz,p}} \tag{3}$$

with the size-independent self-diffusion coefficients of each component as

$$D_{0,p} = \frac{D_{\mathrm{PBC},xx,p} + D_{\mathrm{PBC},yy,p}}{2} \tag{4}$$

and x_p being the mole fraction of component p. The cations and anions should be treated as separate components for a salt mixture.

The OrthoBoXY simulations were performed for the water-modified SIL only. This was due to the decreased mobility for the neat SIL and the long simulation length

required to achieve comparable statistical accuracy for the OrthoBoXY simulations. The simulations were performed under NpT conditions analogous to the cubic systems given above. However, the system sizes were scaled by a factor of 1/10, given the system-size independence of D_0. The resized cubic boxes were equilibrated for 2 ns and then reshaped into orthorhombic box geometries with box length ratios of $L_z/L_x = L_z/L_y \approx 2.7933596497$. The simulation lengths for each system were determined following the suggestions of Busch and Paschek[27], factoring in self-diffusion coefficients obtained from cubic systems as well as the average diameter of the molecules in the mixtures. The simulation lengths are given in Table S3. Simulations were divided into 60 segments, and properties were calculated for each segment to estimate uncertainties using standard statistical methods. Viscosities calculated via the OrthoBoXY method are depicted in Table S4.

Table S3: Total simulation lengths τ_{sim} of OrthoBoXY MD simulations performed under NpT conditions.

[Li][NTf_2]:G3:H_2O	T / K	τ_{sim} / µs
1:1:1	303	17.136
1:1:1	323	6.100

S2.3 Radial Distribution Functions

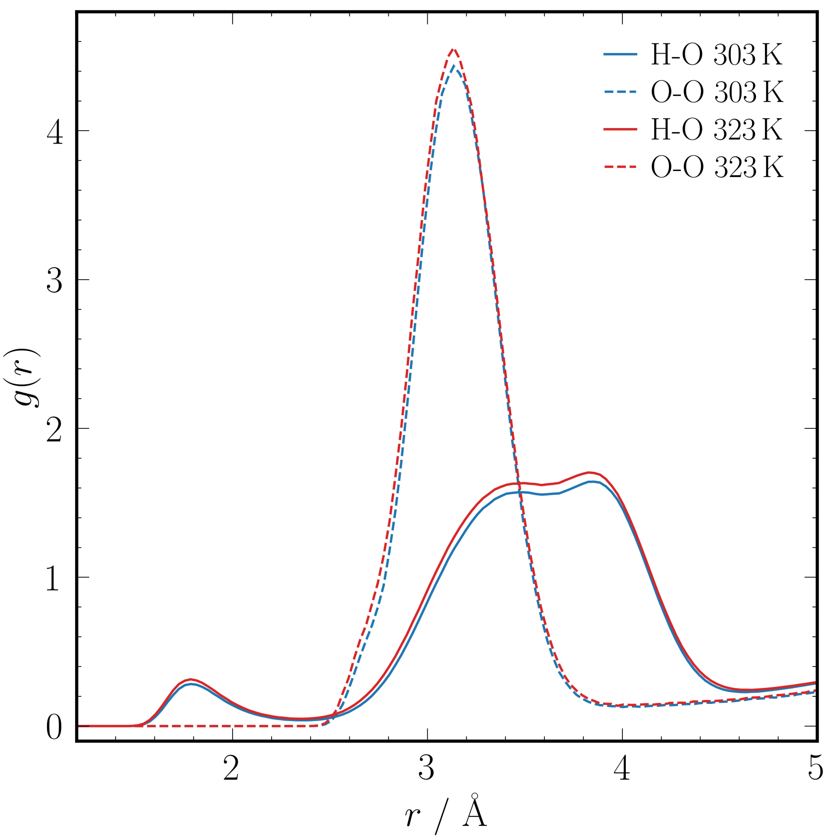

Figure S4: Radial distribution functions of O-O (dashed lines) and H-O (solid lines) between water molecules in [Li][NTf$_2$]:G3:H$_2$O = 1:1:1 at 303 K (blue) and 323 K (red).

S2.4 Self-Diffusion Coefficients

For better comparability between the different systems, the self-diffusion coefficients discussed in this work and summarised in Table S4 all rely on the linear regime of the center-of-mass MSD of the particles in the cubic simulation boxes according to the Einstein formula

$$D_{\mathrm{PBC}} = \frac{1}{6} \lim_{t\to\infty} \frac{\mathrm{d}}{\mathrm{d}t} \left\langle |\vec{r_i}(0) - \vec{r_i}(t)|^2 \right\rangle , \tag{5}$$

where $\vec{r_i}(t)$ represent the position of the center of mass of molecule i at time t. Since D_{PBC} is known to be system-size dependent in cubic simulation boxes with periodic boundary conditions (PBC), the values obtained for D_{PBC} were corrected using the Yeh-Hummer formula[28] from which the system-size independent self-diffusion coefficients D_0 can be obtained as

$$D_0 = D_{\mathrm{PBC}} + \frac{k_{\mathrm{B}} T \xi}{6\pi\eta L}. \tag{6}$$

Here, η denotes the system's viscosity. For this work, the experimental viscosities were employed for the correction. Furthermore, L is the box length, and $\xi \approx 2.837297$.[28]

S2.5 Ionic Conductivity

According to the Einstein-Helfand formalism, the ionic conductivity σ can be computed from the MSD of the collective dipole moment $\vec{M}(t)$ via

$$\sigma = \frac{1}{6Vk_BT} \cdot \frac{\partial}{\partial t} \left\langle |\vec{M}(0) - \vec{M}(t)|^2 \right\rangle \tag{7}$$

with

$$\vec{M}(t) = \sum_{i=1}^{N} q_i \cdot \vec{r_i}(t), \tag{8}$$

where V is the box volume, N denotes the number of particles in the system, and q_i is the charge of each particle.[29] If the charge q_i is expressed as the product of valency and elementary charge, $z_i \cdot e$, Equation 7 can be formed into

$$\sigma = \frac{e^2}{6Vk_BT} \cdot \frac{\partial}{\partial t} \left\langle \sum_{i=1}^{N} \sum_{j=1}^{N} z_i z_j \cdot [\vec{r_i}(0) - \vec{r_i}(t)] \cdot [\vec{r_j}(0) - \vec{r_j}(t)] \right\rangle . \tag{9}$$

The ionic conductivities obtained by employing Equation 9 are listed in Table S4.

Table S4: Physicochemical properties obtained from MD simulations of SIL $[Li(G3)][NTf_2]$ with and without an equimolar amount of water added at 303 K and 323 K.

T	303 K		323 K	
$[Li][NTf_2]$:G3:H_2O	1:1:0	1:1:1	1:1:0	1:1:1
ρ / kg m^{-3}	1426.02 ± 0.20	1390.29 ± 0.08	1402.32 ± 0.25	1368.82 ± 0.06
η / mPa s	—	129.167 ± 0.012	—	46.836 ± 0.004
σ / mS cm^{-1}	0.80 ± 0.08	1.50 ± 0.08	1.76 ± 0.15	2.91 ± 0.17
$D_0([Li]^+)$ / 10^{-12} m^2 s^{-1}	4.28 ± 0.14	9.74 ± 0.12	13.1 ± 0.4	25.32 ± 0.26
D_0(G3) / 10^{-12} m^2 s^{-1}	4.34 ± 0.15	10.02 ± 0.13	13.3 ± 0.4	25.75 ± 0.27
$D_0([NTf_2]^-)$ / 10^{-12} m^2 s^{-1}	3.26 ± 0.12	7.73 ± 0.12	9.32 ± 0.28	19.84 ± 0.26
$D_0(H_2O)$ / 10^{-12} m^2 s^{-1}	—	12.52 ± 0.16	—	29.4 ± 0.4

S2.6 Coordination Numbers

The lithium coordination numbers were calculated from radial distribution functions (RDFs) $g(r)$ via

$$N(r) = 4\pi\rho \int_{0}^{r_\mathrm{c}} g(r) \cdot r^2 \,\mathrm{d}r \tag{10}$$

by integrating the RDFs up to a cut-off radius r_c. This cut-off radius was chosen as the minimum between the first and second peak of the RDF. By this, for the $[Li]^+$-$[NTf_2]^-$ coordination number, a cut-off radius of 5.5 Å was obtained. The cut-off radius for the G3 coordination number of lithium was determined as 3.5 Å. Simultaneously, cut-off radii of 3.0 Å and 2.5 Å were derived for the lithium-oxygen coordination numbers of $[NTf_2]^-$ and G3, respectively. For the site-site RDFs of lithium and the oxygen atoms of the G3 molecules, the shoulder at 3 Å is not taken into account since this peak does not represent coordinating oxygen atoms. Center-of-mass lithium coordination numbers N_COM and lithium-oxygen site-site coordination numbers N_O can be taken from Table S5.

Table S5: Center-of-mass (COM) lithium coordination numbers as well as lithium-oxygen site-site coordination numbers obtained from MD simulations of SIL $[Li(G3)][NTf_2]$ with and without an equimolar amount of water added at 303 K and 323 K.

T	303 K		323 K	
$[Li][NTf_2]$:G3:H_2O	1:1:0	1:1:1	1:1:0	1:1:1
$N_\mathrm{c}^\mathrm{COM}([Li]^+\text{-G3})$	1.00	1.00	1.00	1.00
$N_\mathrm{c}^\mathrm{COM}([Li]^+\text{-}[NTf_2]^-)$	1.30	0.80	1.26	0.78
$N_\mathrm{c}^\mathrm{COM}([Li]^+\text{-}H_2O)$	—	1.00	—	0.99
$N_\mathrm{c}([Li]^+\text{-O[G3]})$	3.77	2.93	3.75	2.92
$N_\mathrm{c}([Li]^+\text{-O}[[NTf_2]^-])$	1.36	0.78	1.31	0.76
$N_\mathrm{c}([Li]^+\text{-O}[H_2O])$	—	1.00	—	0.99

S2.7 Water-water cluster analysis

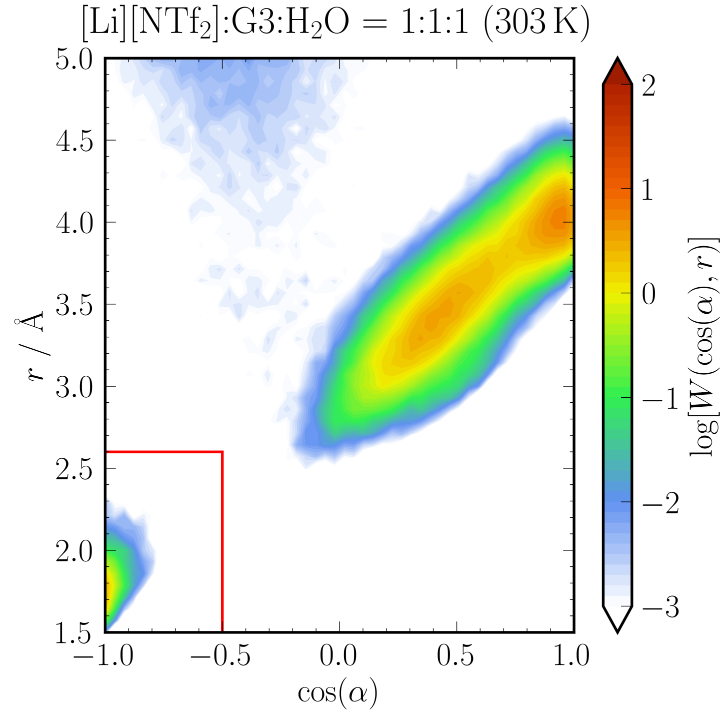

Figure S5: Weighted logarithmic probability density functions $W(\cos(\alpha), r)$ of finding a H-O pair with a hydrogen bond (HB) distance r and a corresponding HB angle $\cos(\alpha)$ at 303 K. The function describes the free energy profile of the interaction between the hydrogen and the oxygen atom of water. The red line represents the chosen cutoff criteria r_c and $\cos(\alpha_c)$ for the HB with the distance cutoff $r_c = 2.6$ Å and the angle cutoff $\cos(\alpha_c) = -0.5$ ($\alpha_c = 120°$).

The percentage of water molecules integrated in water-water clusters can be approximated to 1 % to 2 % from the water-water H-O RDFs (see Figure S4) with the cutoff radius of 2.6 Å, as defined in Figure S5. For this, the number of nearest oxygen atoms to a water hydrogen atom was calculated, excluding the oxygen atom of the same water molecule. However, this neglects the cutoff angle in the definition of an HB in Figure S5. Therefore, the water cluster analysis was carried out using self-written code, considering these distance and angle criteria. The in this way calculated percentages of water molecules being part of water-water clusters are summarized in Table S6.

Table S6: Percentages of water molecules being part of water-water clusters.

[Li][NTf$_2$]:G3:H_2O	T / K	Percentage / %
1:1:1	303	1.80
1:1:1	323	1.84

S2.8 Structural Motifs

Tables S7 and S8 elaborate on the occurrence of different structural motifs in the vicinity of the lithium cations.

Table S7: Occurring combinations of coordinating particles in the vicinity of the $[Li]^+$ cations obtained from MD simulations of the neat SIL $[Li(G3)][NTf_2]$ ($[Li][NTf_2]$:G3:H_2O = 1:1:0) at 303 K and 323 K. The numbers of G3 molecules, and anions in the first solvation sphere are labeled N(G3), and $N([NTf_2]^-)$, respectively. Combinations with an occurrence of less than 1 % were omitted.

N(G3)	$N([NTf_2]^-)$	occurence at 303 K / %	occurence at 323 K / %
1	1	63.7	65.6
1	2	30.2	27.9
2	0	3.0	3.1
0	4	1.5	1.5
1	3	1.2	1.0

Table S8: Occurring combinations of coordinating particles in the vicinity of the $[Li]^+$ cations obtained from MD simulations of the SIL $[Li(G3)][NTf_2]$ with an equimolar amount of water added ($[Li][NTf_2]$:G3:H_2O = 1:1:1) at 303 K and 323 K. The numbers of G3 molecules, water molecules, and anions in the first solvation sphere are labeled N(G3), $N(H_2O)$, and $N([NTf_2]^-)$, respectively. Combinations with an occurrence of less than 1 % were omitted.

N(G3)	$N(H_2O)$	$N([NTf_2]^-)$	occurence at 303 K / %	occurence at 323 K / %
1	1	1	41.9	40.4
1	2	0	22.1	22.4
1	0	1	15.3	16.6
1	1	0	8.4	8.9
1	0	2	6.7	6.2
2	0	0	1.6	1.5

At both temperatures and in both investigated systems, the most abundant structural motif in the lithium coordination sphere consists of a single G3 molecule coordinating to the lithium ion. A more detailed analysis of these chelate complexes, however, reveals pronounced differences between the neat SIL (1:1:0) and the WISIL system (1:1:1) with respect to the specific oxygen atoms of the G3 molecule involved in coordination. These differences are summarized in Table S9, while Figure S6 illustrates the nomenclature of the individual oxygen atoms of the G3 molecule. The oxygen atoms can be grouped into two symmetry-equivalent pairs: the inner oxygen atoms (O2 and O3) and the outer oxygen atoms (O1 and O4). In the neat SIL, all four oxygen atoms exhibit comparable probabilities of forming coordinative bonds to lithium, whereas in the WISIL system,

water molecules preferentially substitute the outer, terminal oxygen atoms rather than the inner oxygen atoms.

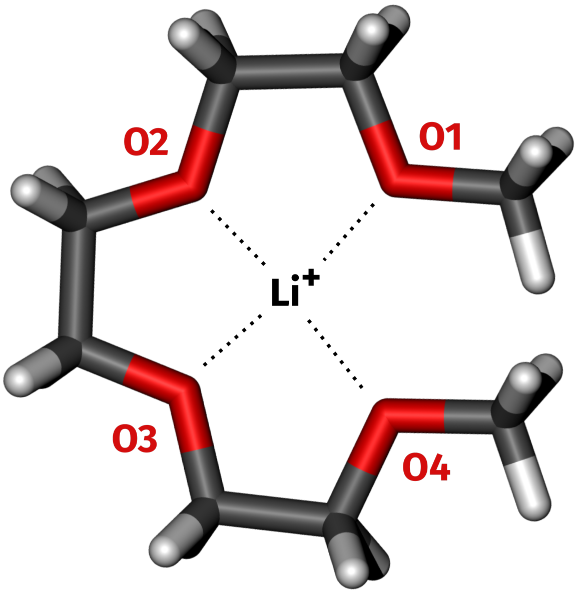

Figure S6: Schematic representation of the G3 molecule in a cyclic conformation that enables chelate coordination to $[Li]^+$. The oxygen atoms are labeled O1 to O4, with O1 and O4 forming one symmetry-equivalent pair and O2 and O3 forming the second, equivalent pair due to the molecular symmetry.

Table S9: Percentage occurrence of coordinative bonding between individual G3 oxygen atoms (O1–O4) and lithium in $[Li(G3)]^+$ complexes containing exactly one G3 molecule. Results are shown for the neat SIL (1:1:0) and the WISIL (1:1:1) systems at 303 K and 323 K.

oxygen atom	1:1:0 (303 K) / %	1:1:0 (323 K) / %	1:1:1 (303 K) / %	1:1:1 (323 K) / %
O1	89.0	89.0	59.1	60.0
O2	88.8	89.0	77.4	77.4
O3	89.0	89.0	77.0	76.6
O4	89.0	88.5	58.0	58.8

References

[1] E. O. Stejskal, J. E. Tanner, *J. Chem. Phys.* **1965**, *42*, 288–292.

[2] I. S. Joung, T. E. I. Cheatham, *J. Phys. Chem. B* **2008**, *112*, 9020–9041.

[3] T. Köddermann, D. Paschek, R. Ludwig, *ChemPhysChem* **2007**, *8*, 2464–2470.

[4] J. Neumann, B. Golub, L.-M. Odebrecht, R. Ludwig, D. Paschek, *J. Chem. Phys.* **2018**, *148*, 193828.

[5] J. Fischer, D. Paschek, A. Geiger, G. Sadowski, *J. Phys. Chem. B* **2008**, *112*, 2388–2398.

[6] J. Fischer, D. Paschek, A. Geiger, G. Sadowski, *J. Phys. Chem. B* **2008**, *112*, 8849–8850.

[7] J. Fischer, D. Paschek, A. Geiger, G. Sadowski, *J. Phys. Chem. B* **2008**, *112*, 13561–13571.

[8] T. Ottallah, S. A. Parandian, S. W. Rick, *J. Chem. Theory Comput.* **2021**, *17*, 315–321.

[9] J. L. F. Abascal, C. Vega, *J. Chem. Phys.* **2005**, *123*, 234505.

[10] E. Lindahl, M. J. Abraham, B. Hess, E. van der Spoel, *GROMACS 2019.6*, Zenodo, **2020**.

[11] H. J. C. Berendsen, D. van der Spoel, R. van Drunen, *Comput. Phys. Commun.* **1995**, *91*, 43–56.

[12] P. Szilárd, M. J. Abraham, C. Kutzner, B. Hess, E. Lindahl in *P. Szilárd, M. J. Abraham, C. Kutzner, B. Hess, E. Lindahl*, *Vol. 8759*, **2015**, pp. 3–27.

[13] L. Martínez, R. Andrade, E. G. Birgin, J. M. Martínez, *J. Comput. Chem.* **2009**, *30*, 2157–2164.

[14] S. Nosé, *Mol. Phys.* **1984**, *52*, 255–268.

[15] S. Nosé, *J. Chem. Phys.* **1984**, *81*, 511–519.

[16] W. G. Hoover, *Phys. Rev. A* **1985**, *31*, 1695–1697.

[17] J. E. Basconi, M. R. Shirts, *J. Chem. Theory Comput.* **2013**, *9*, 2887–2899.

[18] M. Parrinello, A. Rahman, *J. Appl. Phys.* **1981**, *52*, 7182–7190.

[19] T. Darden, D. York, L. Pedersen, *J. Chem. Phys.* **1993**, *98*, 10089–10092.

[20] U. Essmann, L. Perera, M. L. Berkowitz, T. Darden, H. Lee, L. G. Pedersen, *J. Chem. Phys.* **1995**, *103*, 8577–8593.

[21] B. Hess, H. Bekker, H. J. C. Berendsen, J. G. E. M. Fraaije, *J. Comput. Chem.* **1997**, *18*, 1463–1472.

[22] J. T. Bullerjahn, S. von Bülow, M. Heidari, J. Hénin, G. Hummer, *J. Chem. Theory Comput.* **2023**, *19*, 3406–3417.

[23] S. von Bülow, J. T. Bullerjahn, G. Hummer, *J. Chem. Phys.* **2020**, *153*, 021101.

[24] J. Neumann, *MDorado: Collection of Scripts to Analyse Molecular Dynamics Simulations.*, **2022**, https://github.com/Paschek-Lab/MDorado, access date: 05/22/2025.

[25] J. Busch, D. Paschek, *J. Phys. Chem. B* **2023**, *127*, 7983–7987.

[26] J. Busch, D. Paschek, *Phys. Chem. Chem. Phys.* **2024**, *26*, 2907–2914.

[27] J. Busch, D. Paschek, *J. Phys. Chem. B* **2024**, *128*, 1040–1052.

[28] I.-C. Yeh, G. Hummer, *J. Phys. Chem. B* **2004**, *108*, 15873–15879.

[29] C. Schröder, M. Haberler, O. Steinhauser, *J. Chem. Phys.* **2008**, *128*, 134501.

WILEY-VCH

Water in Solvate Ionic Liquids: Preserving Lithium Coordination While Enhancing Ionic Conductivity

Jule Kristin Philipp,*[a] Dietmar Paschek,*[a] Lennart Kruse,[a] Annette-Enrica Surkus,[b] Bennet Austrup,[c] Monika Schönhoff,[c] Ralf Ludwig,*[a,b,d]

This work investigates how the concepts of solvate ionic liquids (SILs) and water-in-salt (WIS) electrolytes can be combined to create hybrid electrolyte systems. We examine the neat SIL [Li(G3)][NTf_2], composed of a solvate cation and an anion, as well as its water-modified analogue containing an equimolar amount of added water, using both molecular dynamics (MD) simulations and experiments. Introducing water markedly reduces the high viscosity of the neat SIL while substantially enhancing ionic conductivity. Structurally, each cationic complex incorporates on average a single water molecule, resulting in highly dispersed water and the absence of extended water networks. We refer to such systems as *water-in-solvate-ionic-liquid* (WISIL) electrolytes. Owing to the strongly coordination-dominated lithium environment, the WISIL retains a wide electrochemical stability window, decreasing only slightly from over 5 V in the neat SIL to 4.9 V at ambient conditions.

Solvate ionic liquids (SILs) represent a distinct class of liquid electrolytes composed of a salt and a molecular solvent capable of forming a stable chelate complex depending on the mixing ratio.[1–4] This unique coordination gives rise to physicochemical properties comparable to those of conventional ionic liquids (ILs).[5–7] In addition, SILs exhibit remarkably high thermal stability, further broadening their applicability in electrochemical systems.[4] By tailoring the composition of a SIL, these properties can be finely tuned, making them promising candidates for safer and more environmentally friendly battery electrolytes.[6–9]

A well-studied class of SILs are equimolar mixtures of lithium bis(trifluoromethanesulfonyl)imide ([Li][NTf_2]) with glyme solvents (H-(CH_2-O-CH_2)$_n$-H), such as triglyme (G3, $n = 4$).[6] Their distinct coordination chemistry leads to well-defined complex cations, e.g., $[Li(G3)]^+$, which exhibit high thermal and electrochemical stability. Nevertheless, the high viscosities and correspondingly low ionic conductivities of these systems limit their practical applicability.[4–6]

Previous studies have shown that dilution with molecular solvents can substantially enhance the ionic conductivity of SILs by lowering viscosity.[6,10–12] However, the nature of the additive plays a crucial role. Nonpolar solvents primarily act as viscosity modifiers without significantly destabilizing the cation–glyme complexes. In contrast to this, Ueno et al. demonstrated that highly polar solvents such as water can interfere with the $[Li]^+$ solvation environment by competing with glyme ligands for coordination.[10,12,13]

Despite these challenges, aqueous electrolytes remain attractive due to their inherent safety and sustainability. However, their narrow electrochemical stability window (ESW) limits their applicability in systems operating at the electrochemical potentials of conventional Li-ion battery electrodes.[10,14] To overcome these limitations, a new class of electrolytes, water-in-salt (WIS) systems, has recently emerged. In these highly concentrated aqueous electrolytes, where the salt content exceeds that of water in both mass and volume, lithium cations remain closely associated with their counterions rather than being fully hydrated. Such water-rich yet ion-dense systems exhibit remarkably broad ESWs of up to ≈ 3 V at room temperature, compared to 1.23 V for conventional aqueous electrolytes, due to the formation of stable interphases that suppress water reduction.[15–20]

In this work, we aim to combine the advantages of SIL and WIS electrolytes by introducing an equimolar amount of water into the SIL [Li(G3)][NTf_2]. Specifically, we address three key questions: (1) How does the addition of water influence the transport and electrochemical properties of the SIL? (2) How does water affect the local solvation structure of lithium cations? and (3) What specific role does water play at such low concentrations in determining the overall physicochemical behavior of the electrolyte?

To address these questions, we investigated the neat SIL ([Li][NTf_2]:G3:H_2O = 1:1:0) and its water-modified analogue containing an equimolar amount of water ([Li][NTf_2]:G3:H_2O = 1:1:1) using both experiments and molecular dynamics (MD) simulations at 303 K and 323 K. Two MD setups were employed: initial simulations in a cubic box, followed by multi-microsecond orthorhombic simulations using the OrthoBoXY approach by Busch and Paschek to determine system-size independent self-diffusion coefficients and viscosities.[22,23] The simulations were complemented by experimental measurements of transport properties, including self-diffusion coefficients from pulsed-field gradient NMR[24] and electrochemical behaviour from cyclic voltam-

[a] J. K. Philipp*, Dr. D. Paschek*, L. Kruse, Prof. R. Ludwig*
Institut für Chemie, Physikalische und Theoretische Chemie, Universität Rostock, Albert-Einstein-Straße 27, D-18059 Rostock, Germany
E-mail: jule.philipp@uni-rostock.de
dietmar.paschek@uni-rostock.de
ralf.ludwig@uni-rostock.de

[b] Dr. A.-E. Surkus, Prof. R. Ludwig*
Leibniz-Institut für Katalyse (LIKAT) an der Universtät Rostock, Albert-Einstein-Straße 29a, D-18059 Rostock, Germany

[c] B. Austrup, Prof. M. Schönhoff
Institute of Physical Chemistry, University of Münster, Corrensstr. 28/30, D-48149 Münster, Germany

[d] Prof. R. Ludwig*
Department LL&M, Universität Rostock, Albert-Einstein-Straße 25, D-18059 Rostock, Germany

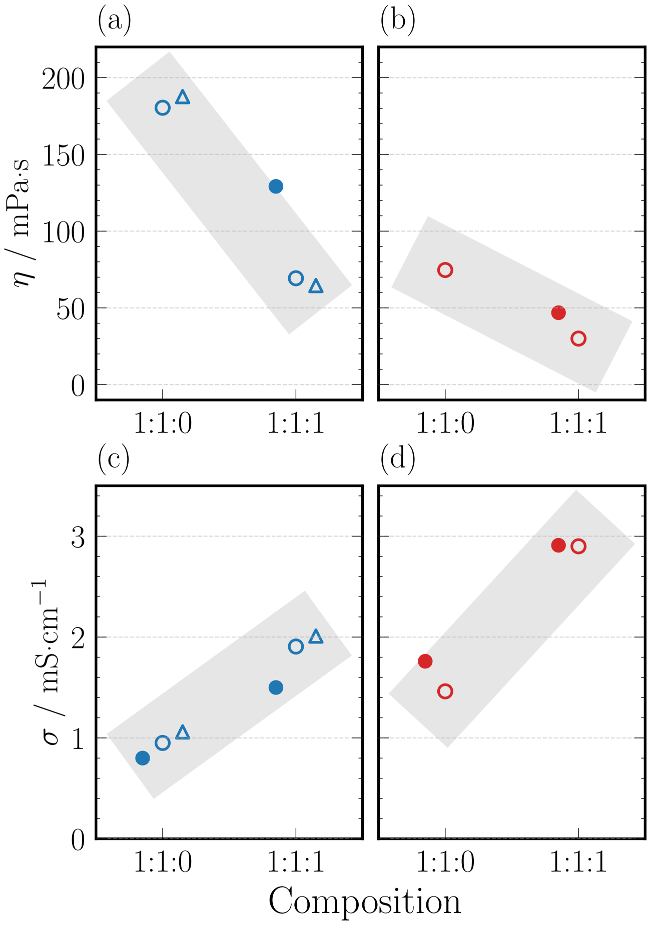

Figure 1. Shear viscosity η (a,b) and ionic conductivity σ (c,d) obtained from experiment (open circles) as well as MD simulations (filled circles) at 303 K (blue) and 323 K (red). Mixture compositions 1:1:0 and 1:1:1 correspond to [Li][NTf_2]:G3:H_2O. Experimental reference data at 303 K (open triangles) are taken from refs.[10,21]. Grey bars serve as visual guides to highlight trends; individual data points are horizontally offset for clarity.

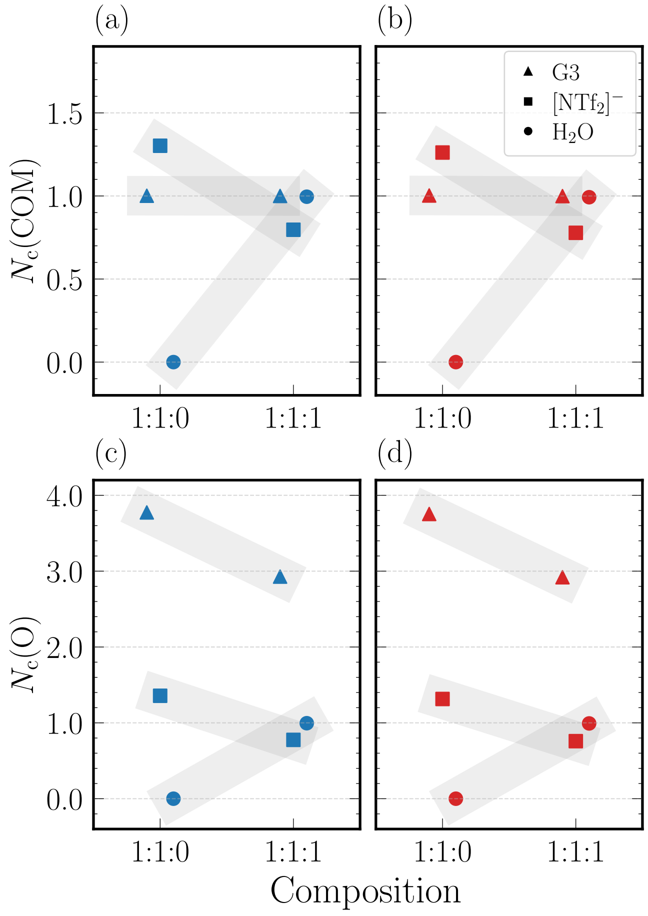

Figure 2. Coordination numbers of $[Li]^+$ with G3 (triangles), $[NTf_2]^-$ (squares), and H_2O (circles) from MD simulations at 303 K (blue) and 323 K (red). N_c(COM) (a,b) denotes the average number of molecules coordinated to $[Li]^+$ based on center-of-mass distances, while oxygen coordination numbers (c,d) represent atomic-level contributions. Mixture compositions 1:1:0 and 1:1:1 correspond to [Li][NTf_2]:G3:H_2O. Grey bars serve as visual guides to highlight trends; individual data points are horizontally offset for clarity.

metry. Detailed descriptions of all experimental procedures and simulation protocols are provided in the Supporting Information.

Experimental densities are well reproduced by the MD simulations for both compositions and temperatures (see SI), providing confidence in the employed molecular model and the microscopic interpretation developed in this study.

Figure 1 summarises the experimental and simulated shear viscosities and ionic conductivities of both systems at the two investigated temperatures. The addition of water has a pronounced effect on the shear viscosity. The neat SIL exhibits a very high viscosity of 180 mPa s at 303 K, which decreases by more than half upon heating to 323 K. Introducing water substantially lowers the viscosity to 69 mPa s at 303 K and to 30 mPa s at 323 K. Viscosities obtained from MD simulations are of the same order of magnitude as the experimental values. However, the high viscosities necessitate long simulation trajectories to achieve acceptable statistical accuracy. Although multi-microsecond simulations still lead to noticeable deviations from experiment, the simulations reproduce the experimental trends well.

The ionic conductivity follows the opposite trend. The neat SIL shows a low conductivity of 0.95 mS cm^{-1} at 303 K, nearly an order of magnitude smaller than that of conventional electrolytes.[25] The water-modified system, however, exhibits an approximately twofold increase in conductivity. At 323 K, the conductivity rises from 1.46 mS cm^{-1} to 2.90 mS cm^{-1}, in near-quantitative agreement with MD simulations. At 303 K, simulations slightly underestimate the conductivity, likely due to the limited ion mobility and associated statistical uncertainty in this highly viscous regime.

Overall, the experimental and simulated results are in very good agreement across the measured properties. This consistency demonstrates that the molecular models accurately capture the macroscopic behavior of these complex electrolytes and supports the reliability of the simulated structural motifs in representing the real liquid systems.

Figure 2 provides insights into the local coordination environment of the lithium cations at both investigated temperatures. Overall, both systems exhibit similar lithium coordination numbers within the studied temperature range, as derived from the data in Table S5. As shown in a previous study, the characteristic coordination motif of the neat SIL remains largely unchanged up to 483 K.[4] In the binary, water-free system, each lithium cation is on average

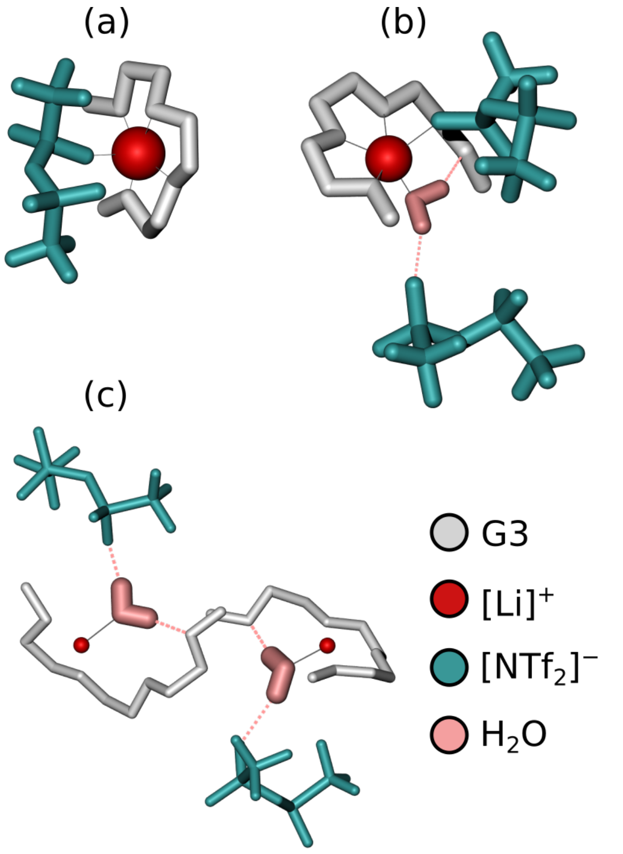

Figure 3. Typical lithium environment for the mixtures with the [Li][NTf$_2$]:G3:H$_2$O composition of (a) 1:1:0 (neat SIL) and (b) 1:1:1, while (c) highlights the spatial separation of water molecules due to the incorporation in complex cations in the water-modified SIL. All snapshots are taken from the MD simulations at 303 K. [NTf$_2$]$^-$ anions, [Li]$^+$ cations, G3 molecules, and H$_2$O molecules are depicted in cyan, red, grey, and pink, respectively. Coordinative bonds are illustrated by black, solid lines, while HBs are shown as pink, dashed lines.

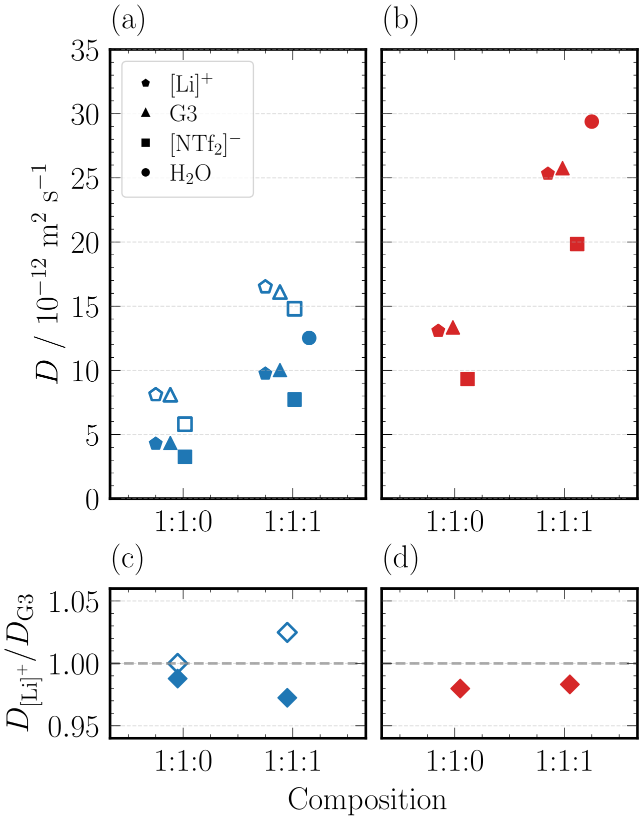

Figure 4. Self-diffusion coefficients of [Li]$^+$ (pentagons), G3 (triangles), [NTf$_2$]$^-$ (squares), and H$_2$O (circles) from MD simulations (filled symbols) at (a) 303 K (blue) and (b) 323 K (red). At 303 K, experimentally obtained values are given as open symbols. (c) and (d) Ratio of self-diffusion coefficients of [Li]$^+$ and G3 obtained from MD simulations (closed symbols) and experiment (open symbols) at 303 K (blue diamonds) and 323 K (red diamonds), respectively. Mixture ratios of 1:1:0 and 1:1:1 correspond to [Li][NTf$_2$]:G3:H$_2$O, respectively. Individual data points are horizontally offset for clarity.

coordinated by a single triglyme molecule, whose four oxygen atoms form a stable chelate complex. The coordination sphere is further complemented by anions, with one or two [NTf$_2$]$^-$ anions contributing one oxygen atom each. A representative snapshot of the most abundant structural motif in the neat SIL is shown in Figure 3a.

Upon addition of an equimolar amount of water, the coordination environment of lithium undergoes subtle but distinct changes. Most lithium cations remain associated with a single triglyme molecule; however, in the majority of cases, only three of its oxygen atoms now directly participate in coordination. The fourth site—most likely one of the two terminal oxygen atoms of the G3 molecule (see Table S8 and Figure S4)—is replaced by a water molecule. This water molecule enters the first coordination shell and simultaneously forms hydrogen bonds (HBs) with both the displaced triglyme oxygen and a nearby anion (Figure 3b). Furthermore, the average number of anions in the first solvation shell decreases to below one, with some anions partially replaced by water. As a result, the overall lithium–oxygen coordination number decreases from 5.1 to 4.7. Additional details on the distribution of structural motifs surrounding [Li]$^+$ are provided in the Supporting Information.

The stability of such complex cations can be assessed by comparing the self-diffusion coefficients of the lithium cation and the coordinating glyme. The ratio $D_{[\mathrm{Li}]^+}/D_{\mathrm{G3}}$ reflects whether lithium and G3 diffuse together, with values near unity indicating stable cationic complexes.[5,12] As shown in Figure 4c/d, this ratio remains close to unity for both the neat and water-modified SIL at 303 K and 323 K in experiment and simulation. The underlying self-diffusion coefficients are demonstrated in Figure 4a for 303 K and in Figure 4b for 323 K. Although experimentally measured self-diffusion coefficients are generally enhanced by a factor of ≈ 2 compared to MD simulation results, they increase with temperature and are consistently higher in the water-containing system, reflecting the reduced viscosity. Yet, the relative mobilities of [Li]$^+$ and G3 remain similar. This finding demonstrates that adding an equimolar amount of water does not significantly disrupt the integrity of the complex cations. While the chosen classical force fields reproduce the trends well, we note that polarizable models are increasingly available and offer promising improvements in accuracy, particularly for ionic liquids[26], and might therefore result in better quantitative agreement of simulation and experiment.

The incorporation of water into the lithium coordination sphere results in the effective spatial separation and dispersion of individual water molecules throughout the system.

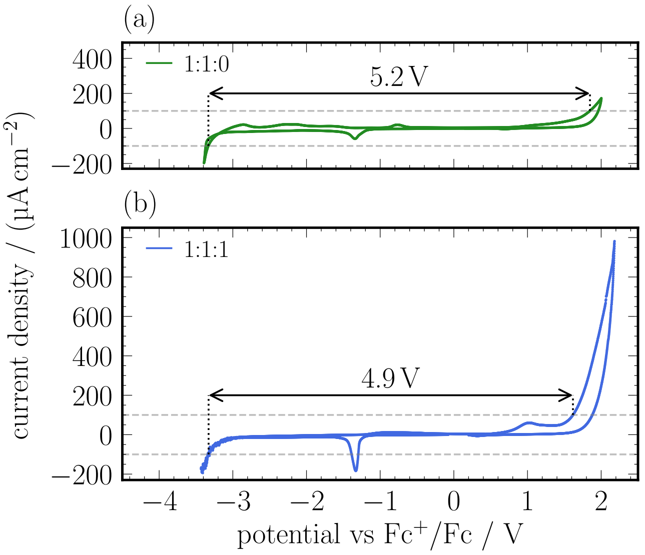

Figure 5. CV curves of (a) the neat SIL (green, measured at 303 K) as well as (b) with an equimolar amount of water added (blue, measured at 296 K). The respective ESWs are denoted above each CV curve. Grey dashed lines mark the limits for determining the respective ESWs at −0.1 mA cm^{-2} and 0.1 mA cm^{-2}. Mixture ratios of 1:1:0 and 1:1:1 correspond to [Li][NTf$_2$]:G3:H_2O, respectively.

This behaviour is illustrated in Figure 3c, where neighbouring complex cations each contain a single, internally coordinated water molecule that is sterically hindered from forming water–water HBs. Consequently, only about below 1.8 % of the water molecules engage in such interactions at both 303 K and 323 K (see SI for more details on computation).

Because the characteristic structural motif of the 1:1:1 system combines features of both disrupted water networks typical of WIS electrolytes and the stable chelate complexes characteristic of SILs, we introduce the term *water-in-solvate-ionic-liquid* (WISIL) electrolyte for this water-modified SIL.

At both temperatures, more than 99 % of all triglyme and water molecules participate in lithium coordination. This coordination-dominated environment is advantageous in SILs, as it minimises the amount of free solvent and thereby reduces solvent degradation at the electrodes.[7] The absence of bulk-like water clusters and the extremely low fraction of uncoordinated solvent molecules suggest an ESW wider than that of conventional aqueous electrolytes and comparable to WIS systems.

Cyclic voltammetry (CV) measurements (see Figure 5) confirm this: the broad ESW, determined at the current density limits of ±0.1 mA cm^{-2}, of the neat SIL of 5.2 V is only slightly reduced to 4.9 V in the WISIL. The 1:1:1 WISIL, however, shows a minor anodic peak at 1.03 V to 1.07 V vs Fc$^+$/Fc attributed to the oxidation of trace amounts of free water at the anode. In addition, a distinct cathodic peak at −1.33 V vs Fc$^+$/Fc emerges. Following Suo et al. and in analogy to classical WIS electrolytes, ~~we assign this cathodic feature primarily to~~ this cathodic feature is likely indicative of the formation of a solid electrolyte interphase (SEI).[15,17] In WIS electrolytes, cathodic reduction of residual surface water molecules generates a locally alkaline environment near the electrode, which promotes anion reduction at potentials close to the hydrogen evolution reaction (HER). The resulting passivating SEI significantly extends the anodic limit of the ESW compared with conventional aqueous electrolytes. A very small cathodic feature at the same potential can also be discerned in the neat SIL, which may also originate from SEI formation. However, further surface analysis is required to confirm the composition of the passivating SEI.

To conclude, experimental data and MD simulations consistently show that adding an equimolar amount of water to the neat SIL [Li(G3)][NTf$_2$] improves the transport properties of the modified SIL by reducing viscosity and thereby doubling ionic conductivity. Structurally, lithium remains coordinated by one G3 molecule, with a single water molecule substituting one donor site and forming hydrogen bonds to the G3 ligand and nearby anions. Despite this modification, the self-diffusion coefficient ratio of [Li]$^+$ and G3 remains close to unity, confirming the persistence of long-lived complex cations. The dispersed, non-associating water molecules enhance transport without disrupting the SIL structure, acting as a molecular lubricant, while maintaining a broad ESW, probably due to the formation of a passivating SEI. Previous studies have shown, however, that the beneficial effects are lost when larger amounts of water are introduced, leading to the breakdown of the complex structure.[10] These insights highlight a narrow but valuable compositional window for optimising SIL-based electrolytes and motivate further studies toward fine-tuning solvation structure and ion mobility for next-generation electrolytes.

Acknowledgements

JKP, DP, and RL thank A. Wilhelms for measuring densities, viscosities, and ionic conductivities. This work has been supported by Deutsche Forschungsgemeinschaft (DFG) with Research Grants LU-506/17-1 (project no. 470038970) and LU-506/18-1 (project no. 517661181).

Conflict of Interest

The authors declare no conflict of interest.

Data Availability Statement

The code of GROMACS is freely available. Input parameter and topology files for the MD simulations can be downloaded from GitHub via
https://github.com/Paschek-Lab/WISIL-G3/

Keywords: Solvate Ionic Liquid • Water-in-Salt Electrolyte • Molecular Dynamics Simulations • Structure • Cluster Formation

References

[1] T. Tamura, K. Yoshida, T. Hachida, M. Tsuchiya, M. Nakamura, Y. Kazue, N. Tachikawa, K. Dokko, M. Watanabe, Chem. Lett. **2010**, 39, 753.
[2] T. Tamura, T. Hachida, K. Yoshida, N. Tachikawa, K. Dokko, M. Watanabe, J. Power Sources **2010**, 195, 6095.
[3] J. K. Philipp, K. Fumino, A. Appelhagen, D. Paschek, R. Ludwig, ChemPhysChem **2025**, 26, e202400991.
[4] J. K. Philipp, L. Kruse, D. Paschek, R. Ludwig, J. Phys. Chem. B **2025**, 129, 5561.
[5] K. Ueno, Electrochemistry **2016**, 84, 674.
[6] K. Ueno, J. Murai, H. Moon, K. Dokko, M. Watanabe, J. Electrochem. Soc. **2016**, 164, A6088.
[7] T. Harte, B. Dharmasiri, Ž. Simon, D. J. Hayne, D. J. Eyckens, L. C. Henderson, J. Mater. Chem. A **2025**, 13, 12746.
[8] D. J. Eyckens, L. C. Henderson, Front. Chem. **2019**, 7, 263.
[9] M. Watanabe, Glyme-based Solvate Ionic Liquids and Their Electrolyte Properties, in K. Yamamoto, H. Nishihara (Editors), Functional Macromolecular Complexes, pages 271–295, Royal Society of Chemistry **2024**.
[10] K. Ueno, J. Murai, K. Ikeda, S. Tsuzuki, M. Tsuchiya, R. Tatara, T. Mandai, Y. Umebayashi, K. Dokko, M. Watanabe, J. Phys. Chem. C **2016**, 120, 15792.
[11] H. Shobukawa, K. Shigenobu, S. Terada, S. Kondou, K. Ueno, K. Dokko, M. Watanabe, Electrochim. Acta **2020**, 353, 136559.
[12] K. Shigenobu, T. Sudoh, J. Murai, K. Dokko, M. Watanabe, K. Ueno, Chem. Rec. **2023**, 23, e202200301.
[13] T. Sudoh, K. Shigenobu, K. Dokko, M. Watanabe, K. Ueno, Phys. Chem. Chem. Phys. **2022**, 24, 14269.
[14] T. F. Burton, R. Jommongkol, Y. Zhu, S. Deebansok, K. Chitbankluai, J. Deng, O. Fontaine, Curr. Opin. Electrochem. **2022**, 35, 101070.
[15] L. Suo, O. Borodin, T. Gao, M. Olguin, J. Ho, X. Fan, C. Luo, C. Wang, K. Xu, Science **2015**, 350, 938.
[16] L. Suo, O. Borodin, W. Sun, X. Fan, C. Yang, F. Wang, T. Gao, Z. Ma, M. Schroeder, A. von Cresce, S. M. Russell, M. Armand, A. Angell, K. Xu, C. Wang, Angew. Chem., Int. Ed. **2016**, 55, 7136.
[17] N. Dubouis, P. Lemaire, B. Mirvaux, E. Salager, M. Deschamps, A. Grimaud, Energy Environ. Sci. **2018**, 11, 3491.
[18] M. Amiri, D. Bélanger, ChemSusChem **2021**, 14, 2487.
[19] A. Tot, L. Zhang, E. J. Berg, P. H. Svensson, L. Kloo, Sci. Rep. **2023**, 13, 2154.
[20] D. Dong, C.-X. Zhao, X. Zhang, C. Wang, Adv. Mater. **2025**, 37, 2418700.
[21] C. Zhang, K. Ueno, A. Yamazaki, K. Yoshida, H. Moon, T. Mandai, Y. Umebayashi, K. Dokko, M. Watanabe, J. Phys. Chem. B **2014**, 118, 5144.
[22] J. Busch, D. Paschek, J. Phys. Chem. B **2023**, 127, 7983.
[23] J. Busch, D. Paschek, J. Phys. Chem. B **2024**, 128, 1040.
[24] M. Holz, S. R. Heil, A. Sacco, Phys. Chem. Chem. Phys. **2000**, 2, 4740.
[25] K. Xu, Chem. Rev. **2004**, 104, 4303.
[26] D. Bedrov, J.-P. Piquemal, O. Borodin, A. D. J. MacKerell, B. Roux, C. Schröder, Chem. Rev. **2019**, 119, 7940.

Entry for the Table of Contents

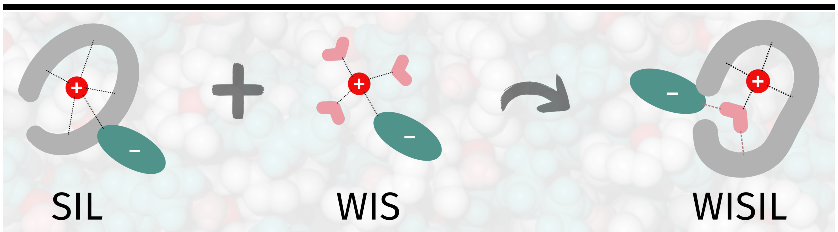

By adding a controlled amount of water to a solvate ionic liquid (SIL), this concept can be bridged with water-in-salt (WIS) electrolytes. Water is inserted into the characteristic cationic complex, leading to *water-in-solvate-ionic-liquid* (WISIL) electrolytes.

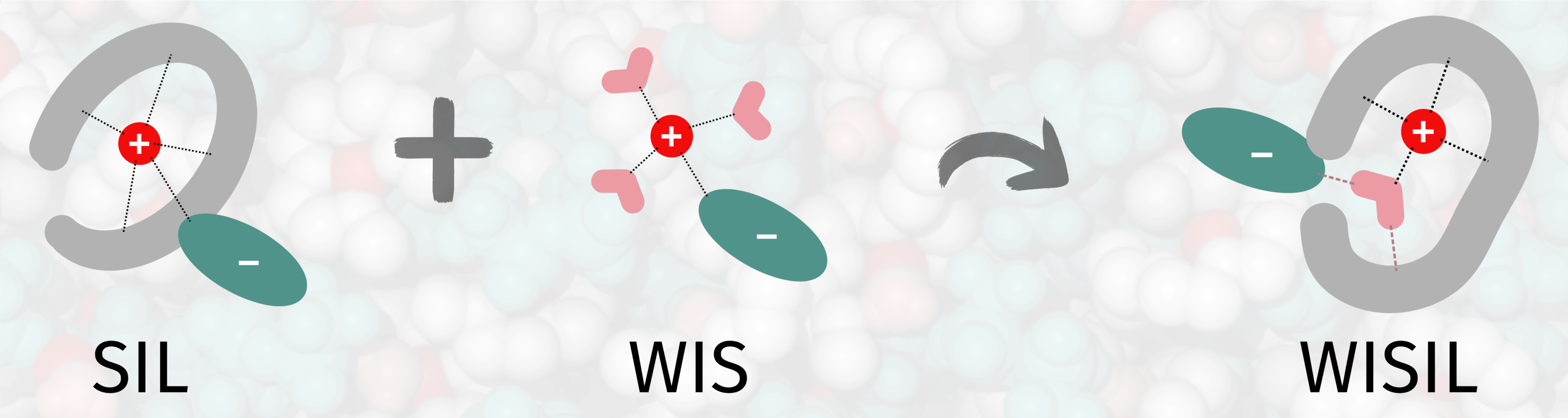
+
+
–
+
+
–
–
+
SIL
WIS
WISIL
